# ﻿Two new species of terrestrial frogs of the *Pristimantisgladiator* complex (Anura, Strabomantidae) from the Ecuadorian Andes, with insights on their biogeography and skull morphology

**DOI:** 10.3897/zookeys.1180.107333

**Published:** 2023-09-26

**Authors:** Juan Pablo Reyes-Puig, Miguel Urgilés-Merchán, Daniela Franco-Mena, Juan M. Guayasamin, Diego Batallas, Carolina Reyes-Puig

**Affiliations:** 1 Unidad de Investigación, Instituto Nacional de Biodiversidad (INABIO), Quito, Ecuador; 2 Fundación EcoMinga; Fundación Oscar Efrén Reyes, Departamento de Ambiente, Baños, Ecuador; 3 Laboratorio de Biología Evolutiva, Colegio de Ciencias Biológicas y Ambientales COCIBA, Universidad San Francisco de Quito USFQ, Quito, Cumbayá, Ecuador; 4 Universidad Complutense de Madrid, Madrid, Spain; 5 Instituto IBIOTROP, Museo de Zoología & Laboratorio de Zoología Terrestre, Colegio de Ciencias Biológicas y Ambientales COCIBA, Universidad San Francisco de Quito USFQ, Quito, Ecuador

**Keywords:** Cryptic species, Ecuadorian Andean slopes, osteology, *Pristimantis* taxonomy

## Abstract

The explosive diversity of rainfrogs (*Pristimantis* spp) reaches its highest levels in the mountains of the Tropical Andes, with remarkable cryptic species mainly in unexplored areas of Ecuador. Based on phylogenetics, morphometric traits, skull osteology and bioacoustics, we describe two new species of *Pristimantis*, previously confused with *Pristimantisgladiator*, that belong to the subgenus Trachyphrynus traditionally known as the *Pristimantismyersi* species group. The two new taxa are closely related, but have allopatric distributions. We discuss the importance of the Quijos and Pastaza River valleys in the diversification along Amazonian slopes of the Ecuadorian Andes.

## ﻿Introduction

The frog genus *Pristimantis* is the largest terrestrial vertebrate radiation in the world, with 603 species formally described to date (Frost 2023). The diversity hotspot of this taxon is concentrated in the Andes of Colombia, Ecuador, and Peru (Reyes-Puig and Mancero 2022; Frost 2023). In Ecuador, we have witnessed a significant increase in the descriptions of new *Pristimantis* species in the last decades, placing this country as the one with the highest number of descriptions per year (Reyes-Puig and Mancero 2022). Many of the new descriptions correspond to morphologically cryptic species (e.g., Hutter and Guayasamin (2015); Reyes-Puig et al. (2019); Páez and Ron (2019); Reyes-Puig et al. (2022)) and recent studies have shown that the undescribed cryptic diversity is still high (Franco-Mena et al. 2023).

Molecular studies have opened the doors to evaluating morphological cryptic diversity by identifying populations that, oftentimes, exhibit evolutionary independence (e.g., reciprocal monophyly, large genetic divergence, low gene flow). A recent molecular study by Franco-Mena et al. (2023) revealed that, within the *Pristimantismyersi* group, now formalized as the subgenus Trachyphrynus, there are numerous candidate species currently hidden under the names of *P.leoni*, *P.hectus*, *P.festae*, *P.gladiator*, and *P.ocreatus*.

In this context, under an integrative approach (e.g. Vieites et al. 2009; Padial et al. 2010), we made a comprehensive review of museum specimens, and new material to address the taxonomic identity of populations currently recognized as *Pristimantisgladiator* in the eastern Andean slopes of Ecuador. We integrated morphological, phylogenetic, acoustic, and osteological approaches, and recognized two new species within the *P.gladiator* complex. We also discuss the biogeographic patterns that seem to be associated with the diversification of these closely-related species.

## ﻿Materials and methods

### ﻿Nomenclature and morphologic analysis

The descriptions of the new species follow the standard format proposed by Lynch and Duellman (1997); the use of diagnostic characters refers to the definitions and illustrations proposed by Duellman and Lehr (2009). We followed the taxonomic arrangements, including species groups, proposed by Heinicke et al. (2007), Hedges et al. (2008), Padial et al. (2014) and Franco-Mena et al. (2023) and summarised by Frost (2023). The collected specimens were euthanised in a benzocaine solution, fixed in formalin at 10%, and preserved in 70% ethanol. The sex and age of the specimens were determined by the identification of secondary sexual characteristics (nuptial pads, males with vocal slits, and body size) and direct gonad inspection through dorsolateral incisions. Morphometric measures were taken with an electronic caliper (precision ± 0.01 mm and approximated to 0.1 mm), following the descriptions by Duellman and Lehr (2009): **SVL** (snout–vent length), **TL** (tibia length), **FL** (foot length, distance from proximal margin of inner metatarsal tubercle to tip of Toe IV), **HaL** (Hand length), **HL** (head length, obliquely from the angle of the jaw to tip of snout), **HW** (head width, at the level of the angle of the jaw), **ED** (eye diameter), **TD** (tympanum diameter), **EN** (eye–nostril distance, straight line distance between the anterior corner of orbit and posterior margin of external nares), **IOD** (interorbital distance), **EW** (upper eyelid width), **IND** (internarial distance), **EN** (eye–nostril distance), **FW** (Finger III width), **TW** (Toe IV width) and, for holotypes, we present additional measurements: forearm length (**FAL**), snout length (**SL**), tarsus length (**TAL**), thigh length (**THL**) and upper arm length (**UAL**). Fingers are numbered pre-axially to postaxial from I–IV. Comparative lengths of Toes III and V were determined when both were adpressed against Toe IV; lengths of Fingers I and II were estimated when adpressed against each other. The colouring patterns in life were described from field notes and colour photographs. To identify morphological differences in the cranial anatomy of the new species and their related congeners, we analized the cranial osteology of females DHMECN 4808, DHMECN 9294, DHMECN 12465, and DHMECN 14447; the samples were prepared with the use of dermestid beetle colonies and subsequently bleached in a sodium dodecyl sulphate solution for 48 hours. The specimens were stored dry inside cotton and paper envelopes. Subsequently, we photographed the cranial samples with a Canon EOS 7D camera. The digital files were coloured with Adobe Photoshop in order to identify and schematize the main cranial differences. Terminology for osteological features follows that described by Duellman and Trueb (1994) and Pramuk (2006). The coordinates and elevation of the type locality were determined, based on coordinates registered by a GPS and collector field notes. Throughout the text, the following abbreviations are used for collectors, photo credits, and associated information: Juan P. Reyes-Puig (**JRP**), Miguel Urgilés M. (**MAUM**), Carolina Reyes-Puig (**CRP**), Daniela Franco Mena (**DFM**). To explore the differences in the morphometric measures extracted from the new species and their related species, we performed a Kruskal-Wallis Rank Sum Test, where the effect of covariation of size (**SVL**) was eliminated through a simple linear regression; thus, the Kruskal-Wallis Test was performed with the residuals of these models. Then, we performed pairwise comparisons between group levels through a Pairwise Wilcoxon Rank Sum Test. Meristic analysis exclude effect of body size (SVL). Type specimens and additional examined material (Appendix 1) are deposited in the Herpetology repository of Instituto Nacional de Biodiversidad, Quito (**DHMECN**), Museo de Zoología of Universidad Tecnológica Indoamérica (**MZUTI**) and Museo de Zoología from Universidad San Francisco de Quito (**ZSFQ**). Examined specimens are listed in Appendix 1.

### ﻿Phylogenetics and genetic distances

Evolutionary relationships were inferred with the same data matrix and criteria described by Franco-Mena et al. (2023), with the addition of a sequence from an individual (ZSFQ 775), which we designated as the holotype of one of the two new species. Pairwise genetic distances were generated from the genetic matrix generated by Franco-Mena et al. (2023), based on the mitochondrial gene 16S, again with the addition of an extra sequence (OR066429-ZSFQ 775).

### ﻿Bioacoustics

Calls were obtained using an Olympus LS-10 Linear PCM field recorder or Digital Record SONY, equipped with a Sennheiser K6–ME 66 unidirectional microphone, at a sample rate of 48 kHz and 24 bit’ resolution and saved in uncompressed WAV format. We record a single calling male of each species on the forest floor, from one to two meters distance, taking file samples of 30 to 45 seconds.

Calls were analized in the software Raven 1.6 (Bioacoustics Research Program 2014), having as settings a Hann window at 90% overlap and 190 samples of DFT size. The parameters analized were: Dominant frequency (**DF**); Number of visible harmonics (**NH**); Harmonics frequencies, corresponding to the series of values that are multiples of the fundamental frequency; Call duration (**CD**); Intervals between calls (**IC**); Call rate (**CR**); Notes/call (**NC**); Note duration (**ND**); Intervals between notes (**IN**); Note rate (**NR**); Pulses/Note (**PN**); Pulse duration (**PD**); Interval between pulses (**IP**); Pulse rate (**PR**). Definitions, terminology and measurements of acoustic parameters follow the definitions summarised in Köhler et al. (2017). In addition, we classified calls into types, as suggested by Emmrich et al. (2020). In the analysis, we follow the note-centered approach, where each note contains subunits or pulses (i.e. call/note/pulse). The figures were processed in R software (R Development Core Team 2022), through the Seewave package version 2.2.0 (Sueur 2018). Audio files in WAV format were imported with the tuneR package version 1.4.1 (Ligges et al. 2018).

## ﻿Results

### ﻿Phylogenetics and genetic distances

**Figure 1.**
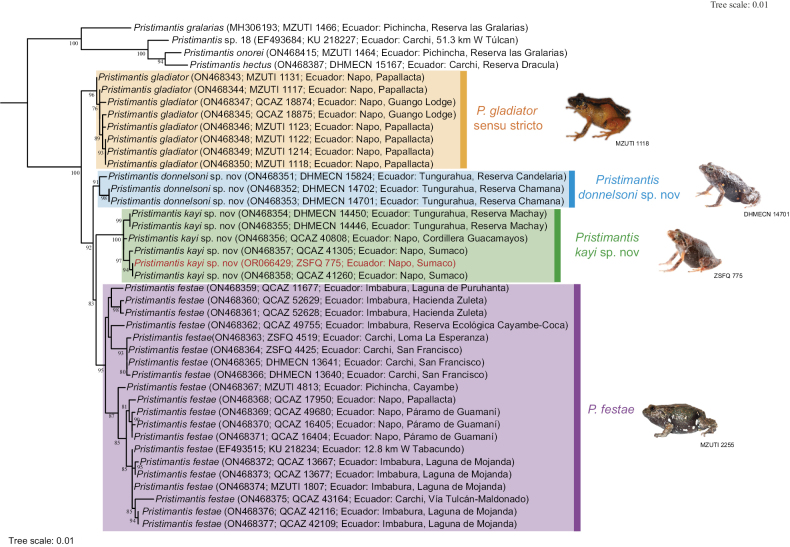
Evolutionary relationships amongst species in the *Pristimantisgladiator* complex, under the Maximum Likelihood inference criterion (modified from Franco-Mena et al. (2023)). Red font indicates the new sequence.

The inferred evolutionary relationships shown in Fig. 1 are the same as those published by Franco-Mena et al. (2023), with additional sequences from an individual (ZSFQ 775) collected at Volcán Sumaco. The new species described below represent Candidate Species 16 and 17, reported by Franco-Mena et al. (2023). Pairwise genetic distances, based on the mitochondrial gene 16S, are summarised in Table 1.

**Table 1.** Pairwise genetic distances between species of the *Pristimantisgladiator* complex and out group of species of *Pristimantismyersi* group. Mean uncorrected *p* distances (gene 16S) between species.

SPECIES	1	2	3	4	5	6	7	8	9	10	11	12	13	14	15	16	17	18	19	20	21	22	23	24	25	26	27	28	29	30	31	32	33	34	35	36	37	38	39	40	41
**1. *Pristimantisonorei* (ON468415)**		0.05	0.05	0.09	0.10	0.10	0.10	0.10	0.10	0.10	0.10	0.10	0.10	0.10	0.10	0.11	0.11	0.11	0.11	0.11	0.11	0.11	0.11	0.11	0.11	0.11	0.11	0.11	0.11	0.11	0.11	0.11	0.11	0.11	0.11	0.11	0.11	0.11	0.11	0.11	0.12
**2. *Pristimantishectus* (ON468387)**	0.05		0.07	0.11	0.12	0.12	0.12	0.12	0.12	0.12	0.12	0.12	0.12	0.12	0.12	0.13	0.13	0.13	0.13	0.13	0.13	0.13	0.13	0.13	0.13	0.13	0.13	0.13	0.13	0.13	0.13	0.13	0.13	0.13	0.13	0.13	0.13	0.13	0.13	0.13	0.14
**3. *Pristimantis* sp. 18 (EF493684)**	0.05	0.07		0.10	0.11	0.11	0.11	0.11	0.11	0.11	0.11	0.11	0.11	0.11	0.11	0.12	0.12	0.12	0.12	0.12	0.12	0.12	0.12	0.12	0.12	0.12	0.12	0.12	0.12	0.12	0.12	0.12	0.12	0.12	0.12	0.12	0.12	0.12	0.12	0.12	0.13
**4. *Pristimantisgralarias* (MH306193)**	0.09	0.11	0.10		0.09	0.09	0.10	0.10	0.10	0.10	0.10	0.10	0.10	0.10	0.10	0.11	0.11	0.10	0.11	0.11	0.11	0.10	0.11	0.10	0.11	0.10	0.10	0.10	0.10	0.10	0.11	0.11	0.11	0.11	0.11	0.11	0.11	0.11	0.11	0.11	0.12
**5. *Pristimantisgladiator* (ON468343)**	0.10	0.12	0.11	0.09		0.00	0.00	0.00	0.00	0.00	0.00	0.00	0.02	0.02	0.02	0.03	0.03	0.02	0.03	0.03	0.03	0.02	0.03	0.02	0.03	0.02	0.02	0.02	0.02	0.02	0.03	0.03	0.03	0.03	0.03	0.03	0.03	0.03	0.03	0.03	0.03
**6. *Pristimantisgladiator* (ON468344)**	0.10	0.12	0.11	0.09	0.00		0.00	0.00	0.00	0.00	0.00	0.00	0.02	0.02	0.02	0.03	0.03	0.03	0.03	0.03	0.03	0.02	0.03	0.03	0.03	0.03	0.02	0.02	0.02	0.02	0.03	0.03	0.03	0.03	0.03	0.03	0.03	0.03	0.03	0.03	0.04
**7. *Pristimantisgladiator* (ON468347)**	0.10	0.12	0.11	0.10	0.00	0.00		0.00	0.00	0.00	0.00	0.00	0.02	0.02	0.02	0.03	0.03	0.03	0.03	0.03	0.03	0.03	0.03	0.03	0.03	0.03	0.03	0.03	0.03	0.03	0.03	0.03	0.03	0.03	0.03	0.03	0.03	0.03	0.03	0.03	0.04
**8. *Pristimantisgladiator* (ON468350)**	0.10	0.12	0.11	0.10	0.00	0.00	0.00		0.00	0.00	0.00	0.00	0.02	0.02	0.02	0.03	0.03	0.03	0.03	0.03	0.03	0.03	0.03	0.03	0.03	0.03	0.03	0.03	0.03	0.03	0.03	0.03	0.03	0.03	0.03	0.03	0.03	0.03	0.03	0.03	0.04
**9. *Pristimantisgladiator* (ON468349)**	0.10	0.12	0.11	0.10	0.00	0.00	0.00	0.00		0.00	0.00	0.00	0.02	0.02	0.02	0.03	0.03	0.03	0.03	0.03	0.03	0.03	0.03	0.03	0.03	0.03	0.03	0.03	0.03	0.03	0.03	0.03	0.03	0.03	0.03	0.03	0.03	0.03	0.03	0.03	0.04
**10. *Pristimantisgladiator* (ON468348)**	0.10	0.12	0.11	0.10	0.00	0.00	0.00	0.00	0.00		0.00	0.00	0.02	0.02	0.02	0.03	0.03	0.03	0.03	0.03	0.03	0.03	0.03	0.03	0.03	0.03	0.03	0.03	0.03	0.03	0.03	0.03	0.03	0.03	0.03	0.03	0.03	0.03	0.03	0.03	0.04
**11. *Pristimantisgladiator* (ON468346)**	0.10	0.12	0.11	0.10	0.00	0.00	0.00	0.00	0.00	0.00		0.00	0.02	0.02	0.02	0.03	0.03	0.03	0.03	0.03	0.03	0.03	0.03	0.03	0.03	0.03	0.03	0.03	0.03	0.03	0.03	0.03	0.03	0.03	0.03	0.03	0.03	0.03	0.03	0.03	0.04
**12. *Pristimantisgladiator* (ON468345)**	0.10	0.12	0.11	0.10	0.00	0.00	0.00	0.00	0.00	0.00	0.00		0.02	0.02	0.02	0.03	0.03	0.03	0.03	0.03	0.03	0.03	0.03	0.03	0.03	0.03	0.03	0.03	0.03	0.03	0.03	0.03	0.03	0.03	0.03	0.03	0.03	0.03	0.03	0.03	0.04
**13. *Pristimantisdonnelsoni* sp. nov. (ON468351)**	0.10	0.12	0.11	0.10	0.02	0.02	0.02	0.02	0.02	0.02	0.02	0.02		0.01	0.01	0.02	0.02	0.02	0.02	0.02	0.02	0.02	0.02	0.02	0.02	0.02	0.02	0.02	0.02	0.02	0.02	0.02	0.02	0.02	0.02	0.02	0.02	0.02	0.02	0.02	0.03
**14. *Pristimantisdonnelsoni* sp. nov. (ON468352)**	0.10	0.12	0.11	0.10	0.02	0.02	0.02	0.02	0.02	0.02	0.02	0.02	0.01		0.00	0.02	0.02	0.02	0.02	0.02	0.02	0.02	0.02	0.02	0.02	0.02	0.02	0.02	0.02	0.02	0.02	0.02	0.02	0.02	0.02	0.02	0.02	0.02	0.02	0.02	0.03
**15. Pristimantisdonnelsoni sp. nov. (ON468353)**	0.10	0.12	0.11	0.10	0.02	0.02	0.02	0.02	0.02	0.02	0.02	0.02	0.01	0.00		0.02	0.02	0.02	0.02	0.02	0.02	0.02	0.02	0.02	0.02	0.02	0.02	0.02	0.02	0.02	0.02	0.02	0.02	0.02	0.02	0.02	0.02	0.02	0.02	0.02	0.03
**16. *Pristimantiskayi* sp. nov. (ON468355)**	0.11	0.13	0.12	0.11	0.03	0.03	0.03	0.03	0.03	0.03	0.03	0.03	0.02	0.02	0.02		0.00	0.00	0.01	0.01	0.01	0.02	0.03	0.03	0.03	0.03	0.02	0.02	0.02	0.02	0.03	0.03	0.03	0.03	0.03	0.03	0.03	0.03	0.03	0.03	0.04
**17. *Pristimantiskayi* sp. nov. (ON468354)**	0.11	0.13	0.12	0.11	0.03	0.03	0.03	0.03	0.03	0.03	0.03	0.03	0.02	0.02	0.02	0.00		0.00	0.01	0.01	0.01	0.02	0.03	0.03	0.03	0.03	0.02	0.02	0.02	0.02	0.03	0.03	0.03	0.03	0.03	0.03	0.03	0.03	0.03	0.03	0.04
**18. *Pristimantiskayi* sp. nov. (ON468356)**	0.11	0.13	0.12	0.10	0.02	0.03	0.03	0.03	0.03	0.03	0.03	0.03	0.02	0.02	0.02	0.00	0.00		0.00	0.00	0.00	0.02	0.03	0.02	0.03	0.02	0.02	0.02	0.02	0.02	0.03	0.03	0.03	0.03	0.03	0.03	0.03	0.03	0.03	0.03	0.03
**19. *Pristimantiskayi* sp. nov. (ON468357)**	0.11	0.13	0.12	0.11	0.03	0.03	0.03	0.03	0.03	0.03	0.03	0.03	0.02	0.02	0.02	0.01	0.01	0.00		0.00	0.00	0.03	0.03	0.03	0.03	0.03	0.03	0.03	0.03	0.03	0.03	0.03	0.03	0.03	0.03	0.03	0.03	0.03	0.03	0.03	0.04
**20. *Pristimantiskayi* sp. nov. (ON468358)**	0.11	0.13	0.12	0.11	0.03	0.03	0.03	0.03	0.03	0.03	0.03	0.03	0.02	0.02	0.02	0.01	0.01	0.00	0.00		0.00	0.03	0.03	0.03	0.03	0.03	0.03	0.03	0.03	0.03	0.03	0.03	0.03	0.03	0.03	0.03	0.03	0.03	0.03	0.03	0.04
**21. *Pristimantiskayi* sp. nov. (OR066429)**	0.11	0.13	0.12	0.11	0.03	0.03	0.03	0.03	0.03	0.03	0.03	0.03	0.02	0.02	0.02	0.01	0.01	0.00	0.00	0.00		0.03	0.03	0.03	0.03	0.03	0.03	0.03	0.03	0.03	0.03	0.03	0.03	0.03	0.03	0.03	0.03	0.03	0.03	0.03	0.04
**22. *Pristimantisfestae* (ON468362)**	0.11	0.13	0.12	0.10	0.02	0.02	0.03	0.03	0.03	0.03	0.03	0.03	0.02	0.02	0.02	0.02	0.02	0.02	0.03	0.03	0.03		0.01	0.01	0.01	0.01	0.01	0.01	0.01	0.02	0.02	0.02	0.02	0.02	0.02	0.02	0.02	0.02	0.02	0.02	0.03
**23. *Pristimantisfestae* (ON468363)**	0.11	0.13	0.12	0.11	0.03	0.03	0.03	0.03	0.03	0.03	0.03	0.03	0.02	0.02	0.02	0.03	0.03	0.03	0.03	0.03	0.03	0.01		0.00	0.00	0.00	0.01	0.01	0.01	0.02	0.02	0.02	0.02	0.02	0.02	0.02	0.02	0.02	0.02	0.02	0.03
**24. *Pristimantisfestae* (ON468364)**	0.11	0.13	0.12	0.10	0.02	0.03	0.03	0.03	0.03	0.03	0.03	0.03	0.02	0.02	0.02	0.03	0.03	0.02	0.03	0.03	0.03	0.01	0.00		0.00	0.00	0.01	0.01	0.01	0.02	0.02	0.02	0.02	0.02	0.02	0.02	0.02	0.02	0.02	0.02	0.03
**25. *Pristimantisfestae* (ON468366)**	0.11	0.13	0.12	0.11	0.03	0.03	0.03	0.03	0.03	0.03	0.03	0.03	0.02	0.02	0.02	0.03	0.03	0.03	0.03	0.03	0.03	0.01	0.00	0.00		0.00	0.01	0.01	0.01	0.02	0.02	0.02	0.02	0.02	0.02	0.02	0.02	0.02	0.02	0.02	0.03
**26. *Pristimantisfestae* (ON468365)**	0.11	0.13	0.12	0.10	0.02	0.03	0.03	0.03	0.03	0.03	0.03	0.03	0.02	0.02	0.02	0.03	0.03	0.02	0.03	0.03	0.03	0.01	0.00	0.00	0.00		0.01	0.01	0.01	0.02	0.02	0.02	0.02	0.02	0.02	0.02	0.02	0.02	0.02	0.02	0.03
**27. *Pristimantisfestae* (ON468359)**	0.11	0.13	0.12	0.10	0.02	0.02	0.03	0.03	0.03	0.03	0.03	0.03	0.02	0.02	0.02	0.02	0.02	0.02	0.03	0.03	0.03	0.01	0.01	0.01	0.01	0.01		0.01	0.01	0.01	0.02	0.02	0.02	0.02	0.02	0.02	0.02	0.02	0.02	0.02	0.03
**28. *Pristimantisfestae* (ON468360)**	0.11	0.13	0.12	0.10	0.02	0.02	0.03	0.03	0.03	0.03	0.03	0.03	0.02	0.02	0.02	0.02	0.02	0.02	0.03	0.03	0.03	0.01	0.01	0.01	0.01	0.01	0.01		0.00	0.02	0.02	0.02	0.02	0.02	0.02	0.02	0.02	0.02	0.02	0.02	0.03
**29. *Pristimantisfestae* (ON468361)**	0.11	0.13	0.12	0.10	0.02	0.02	0.03	0.03	0.03	0.03	0.03	0.03	0.02	0.02	0.02	0.02	0.02	0.02	0.03	0.03	0.03	0.01	0.01	0.01	0.01	0.01	0.01	0.00		0.02	0.02	0.02	0.02	0.02	0.02	0.02	0.02	0.02	0.02	0.02	0.03
**30. *Pristimantisfestae* (ON468367)**	0.11	0.13	0.12	0.10	0.02	0.02	0.03	0.03	0.03	0.03	0.03	0.03	0.02	0.02	0.02	0.02	0.02	0.02	0.03	0.03	0.03	0.02	0.02	0.02	0.02	0.02	0.01	0.02	0.02		0.01	0.01	0.01	0.01	0.01	0.01	0.01	0.01	0.01	0.01	0.02
**31. *Pristimantisfestae* (ON468368)**	0.11	0.13	0.12	0.11	0.03	0.03	0.03	0.03	0.03	0.03	0.03	0.03	0.02	0.02	0.02	0.03	0.03	0.03	0.03	0.03	0.03	0.02	0.02	0.02	0.02	0.02	0.02	0.02	0.02	0.01		0.00	0.00	0.00	0.01	0.01	0.01	0.01	0.01	0.01	0.01
**32. *Pristimantisfestae* (ON468371)**	0.11	0.13	0.12	0.11	0.03	0.03	0.03	0.03	0.03	0.03	0.03	0.03	0.02	0.02	0.02	0.03	0.03	0.03	0.03	0.03	0.03	0.02	0.02	0.02	0.02	0.02	0.02	0.02	0.02	0.01	0.00		0.00	0.00	0.00	0.01	0.01	0.00	0.01	0.01	0.01
**33. *Pristimantisfestae* (ON468370)**	0.11	0.13	0.12	0.11	0.03	0.03	0.03	0.03	0.03	0.03	0.03	0.03	0.02	0.02	0.02	0.03	0.03	0.03	0.03	0.03	0.03	0.02	0.02	0.02	0.02	0.02	0.02	0.02	0.02	0.01	0.00	0.00		0.00	0.01	0.01	0.01	0.01	0.01	0.01	0.01
**34. *Pristimantisfestae* (ON468369)**	0.11	0.13	0.12	0.11	0.03	0.03	0.03	0.03	0.03	0.03	0.03	0.03	0.02	0.02	0.02	0.03	0.03	0.03	0.03	0.03	0.03	0.02	0.02	0.02	0.02	0.02	0.02	0.02	0.02	0.01	0.00	0.00	0.00		0.01	0.01	0.01	0.01	0.01	0.01	0.01
**35. *Pristimantisfestae* (EF493515)**	0.11	0.13	0.12	0.11	0.03	0.03	0.03	0.03	0.03	0.03	0.03	0.03	0.02	0.02	0.02	0.03	0.03	0.03	0.03	0.03	0.03	0.02	0.02	0.02	0.02	0.02	0.02	0.02	0.02	0.01	0.01	0.00	0.01	0.01		0.00	0.00	0.00	0.00	0.00	0.01
**36. *Pristimantisfestae* (ON468373)**	0.11	0.13	0.12	0.11	0.03	0.03	0.03	0.03	0.03	0.03	0.03	0.03	0.02	0.02	0.02	0.03	0.03	0.03	0.03	0.03	0.03	0.02	0.02	0.02	0.02	0.02	0.02	0.02	0.02	0.01	0.01	0.01	0.01	0.01	0.00		0.00	0.00	0.00	0.00	0.01
**37. *Pristimantisfestae* (ON468372)**	0.11	0.13	0.12	0.11	0.03	0.03	0.03	0.03	0.03	0.03	0.03	0.03	0.02	0.02	0.02	0.03	0.03	0.03	0.03	0.03	0.03	0.02	0.02	0.02	0.02	0.02	0.02	0.02	0.02	0.01	0.01	0.01	0.01	0.01	0.00	0.00		0.00	0.00	0.00	0.01
**39. *Pristimantisfestae* (ON468374)**	0.11	0.13	0.12	0.11	0.03	0.03	0.03	0.03	0.03	0.03	0.03	0.03	0.02	0.02	0.02	0.03	0.03	0.03	0.03	0.03	0.03	0.02	0.02	0.02	0.02	0.02	0.02	0.02	0.02	0.01	0.01	0.00	0.01	0.01	0.00	0.00	0.00		0.00	0.00	0.01
**40. *Pristimantisfestae* (ON468376)**	0.11	0.13	0.12	0.11	0.03	0.03	0.03	0.03	0.03	0.03	0.03	0.03	0.02	0.02	0.02	0.03	0.03	0.03	0.03	0.03	0.03	0.02	0.02	0.02	0.02	0.02	0.02	0.02	0.02	0.01	0.01	0.01	0.01	0.01	0.00	0.00	0.00	0.00		0.00	0.01
**41. *Pristimantisfestae* (ON468377)**	0.11	0.13	0.12	0.11	0.03	0.03	0.03	0.03	0.03	0.03	0.03	0.03	0.02	0.02	0.02	0.03	0.03	0.03	0.03	0.03	0.03	0.02	0.02	0.02	0.02	0.02	0.02	0.02	0.02	0.01	0.01	0.01	0.01	0.01	0.00	0.00	0.00	0.00	0.00		0.01
**42. *Pristimantisfestae* (ON468375)**	0.12	0.14	0.13	0.12	0.03	0.04	0.04	0.04	0.04	0.04	0.04	0.04	0.03	0.03	0.03	0.04	0.04	0.03	0.04	0.04	0.04	0.03	0.03	0.03	0.03	0.03	0.03	0.03	0.03	0.02	0.01	0.01	0.01	0.01	0.01	0.01	0.01	0.01	0.01	0.01	

### ﻿Systematics

#### ﻿Species account

##### ﻿ Pristimantis donnelsoni

Taxon classificationAnimaliaAnuraStrabomantidae

Juan Pablo Reyes-Puig, Miguel Urgilés-Merchán, Carolina Reyes-Puig, Daniela Franco-Mena, Diego Batallas & Juan M. Guayasamin sp. nov.

85F826E5-8E0F-5EDA-BF3A-A1FF49A9BDDE

https://zoobank.org/B05D8CB1-738F-4B33-A4C8-7AA468510BED

Figs 1 , 2 , 3 , 4 , 5 , 6 , 7 , 8

###### Type material.

**Figure 2.**
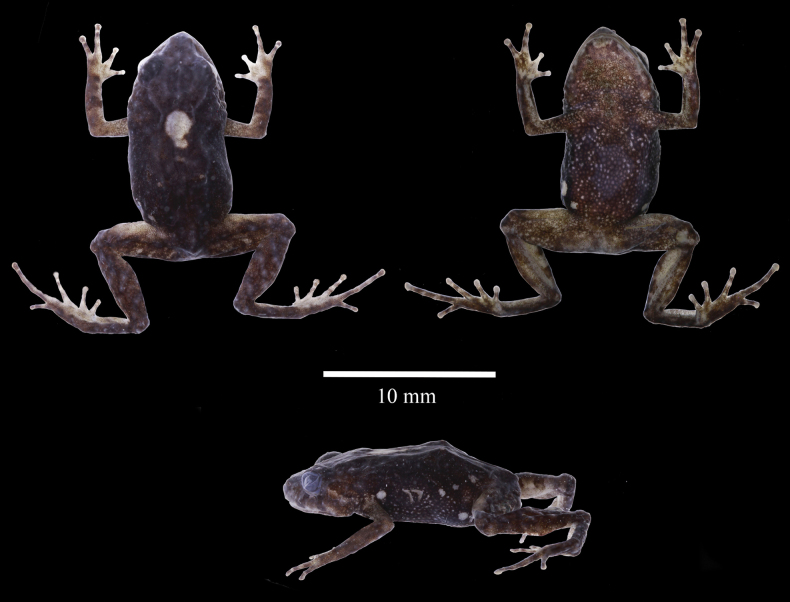
*Pristimantisdonnelsoni* sp. nov. in ethanol. Adult female, holotype, DHMECN 14701, in dorsal, ventral and lateral views. Photographs by JPRP.

**Figure 3.**
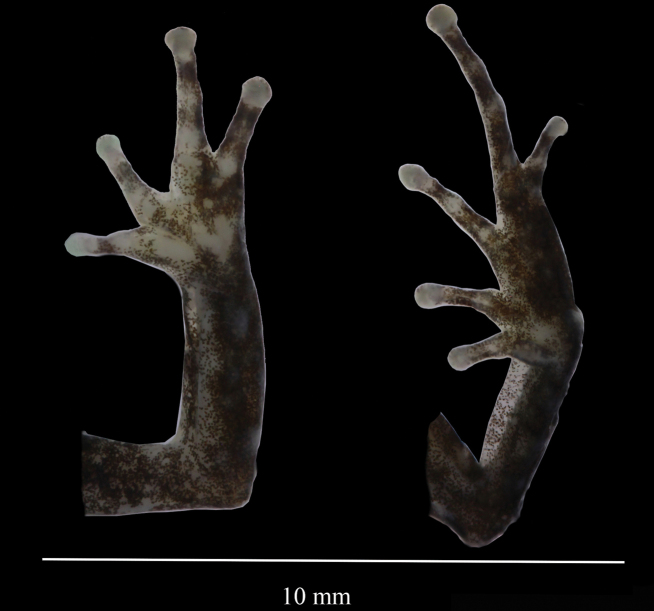
Ventral view of left hand and foot of *Pristimantisdonnelsoni* sp. nov. Adult female, holotype, DHMECN 14701. Photographs by JPRP.

**Figure 4.**
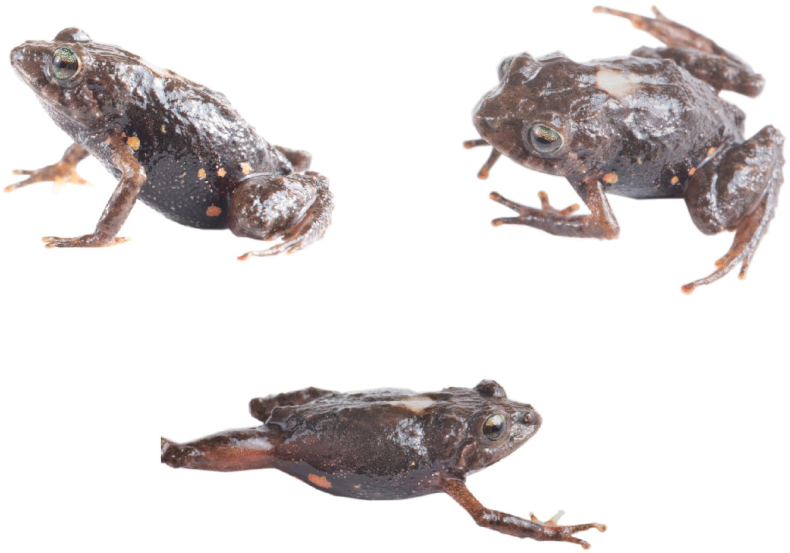
Lateral views of *Pristimantisdonnelsoni* sp. nov. in life. Adult female, holotype DHMECN 14701. LRC: 15.42 mm. Photographs by Frank Pichardo.

**Figure 5.**
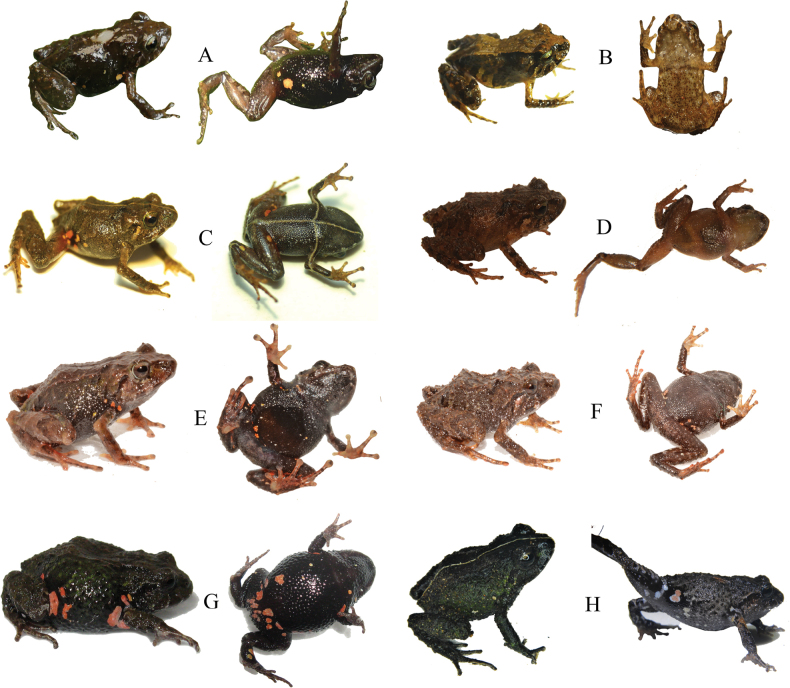
Dorsolateral and ventral views in life of *Pristimantisgladiator* species complex and related species **A***Pristimantisdonnelsoni* sp. nov. female, DHMECN 14701, holotype, LRC: 15.42 mm **B***Pristimantisdonnelsoni* sp. nov. male, DHMECN 18854, LRC: 13.66 mm from Cerro Candelaria **C***Pristimantiskayi* sp. nov. female, ZSFQ 0775, holotype, LRC: 19.7 mm **D***Pristimantiskayi* sp. nov. male, DHMECN 14450 LRC: 13.58 mm, from Reserva Machay **E***Pristimantisgladiator* sensu stricto female, DHMECN 12463, LRC: 20.38 mm **F***Pristimantisgladiator* sensu stricto, male DHMECN 12474, LRC: 16.87 mm; from Río Quijos drainage **G***Pristimantisfestae* female, DHMECN 16280, LRC: 20.19 mm **H***Pristimantisfestae*, male (left), DHMECN 16837, LRC: 16.50 mm; female (right), DHMECN 16836, LRC: 19.80 mm; Photographers: JPRP (**A, B**), MYM (**D, E, F, H**); Patricia Bejarano (**G**); José Vieira (**C**).

**Figure 6.**
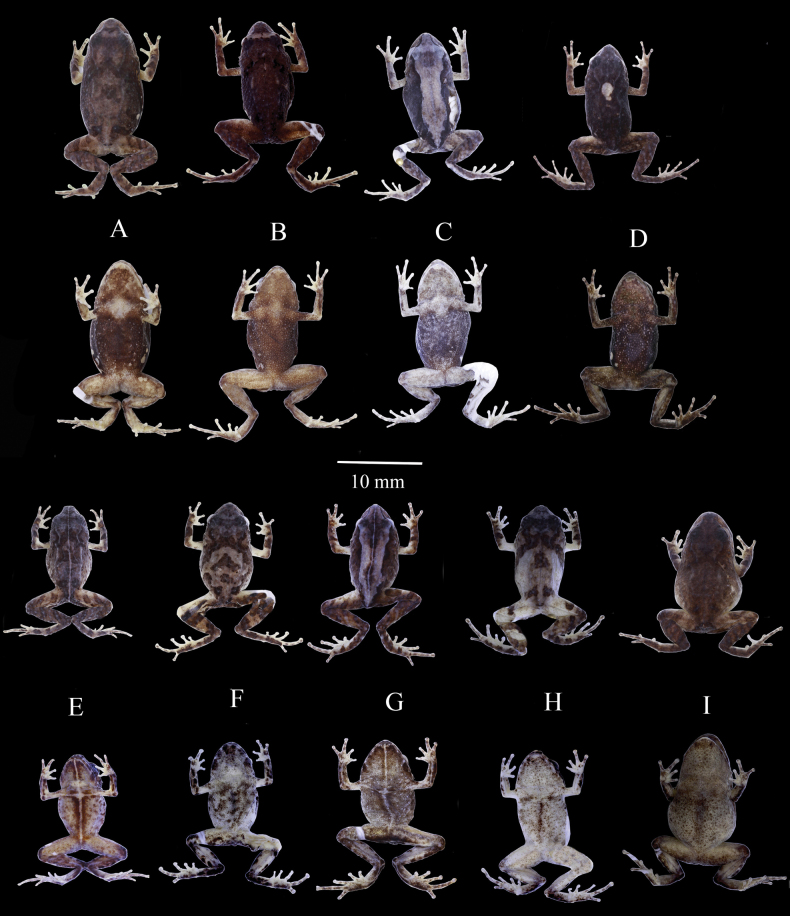
*Pristimantisdonnelsoni* sp. nov. dorsal and ventral views of the preserved types series **A**DHMECN 4779, female **B**DHMECN 4782, female **C** female, ZSFQ 1097 **D** holotype, 14701, female **E**DHMECN 4781, male **F**DHMECN 16613, male **G**DHMECN 16610, male **H**ZSFQ 1098, male **I**DHMECN 4778, male. Photographs by JPRP and David Brito Zapata.

**Figure 7.**
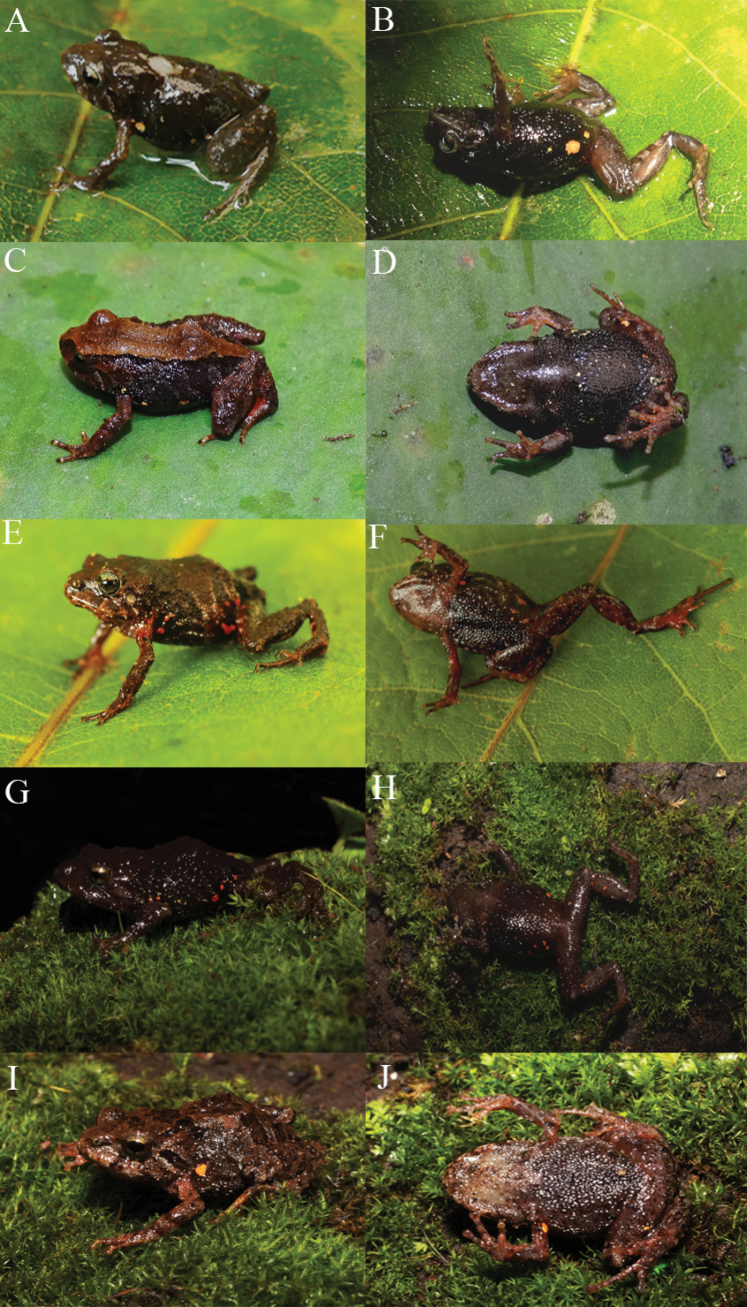
Life dorsal and ventral views of *Pristimantisdonnelsoni* sp. nov., in situ **A, B** holotype male DHMECN 14701, LRC: 15.42 mm, from Chamana Reserve **C, D** paratype female DHMECN 13321, LRC: 15.47 mm, from Cerro Candelaria **E, F** paratype female DHMECN 16175, LRC:13.71 mm, from Cerro Candelaria **G, H** paratype female DHMECN 18159, LRC:18.72 mm, from Hacienda Guamag **I, J** adult male DHMECN 18185, LRC:14.26 mm, from Finca Palmonte. Photographs by JPRP.

***Holotype*.**DHMECN 14701, adult female (Figs 2–7), collected by CRP, JPRP, and DFM from Reserva Chamana, eastern slopes of Tungurahua Volcano, Ulba, Baños, Tungurahua, Ecuador (1.4272°S, 78.3967°W; 3,028 m alt.) on 13 August 2018.

***Paratypes*.** (22, 13 ♂ / 9 ♀, Fig. 6): ZSFQ 1087, adult female collected by CRP, JPRP and DFM from Reserva Chamana, Ulba, Baños, Tungurahua, Ecuador (1.421°S, 78.3866°W; 2909 m alt.) on 11 August 2018. ZSFQ 1090 and 1096, adult females collected by CRP, JPRP, and DFM from Reserva Chamana, Ulba, Baños, Tungurahua, Ecuador (1.4256°S, 78.3854°W; 2977 m alt.) on 12 August 2018. ZSFQ 1100, adult male collected by CRP, JPRP, and DFM from Reserva Chamana, Ulba, Baños, Tungurahua, Ecuador (1.4273°S, 78.3853°W; 3000 m alt.) on 12 August 2018. ZSFQ 1103 and 1105, adult males collected with the same data as ZSFQ 1090. DHMECN 16606, adult male collected by JPRP from Reserva Chamana, Ulba, Baños de Agua Santa, Tungurahua, Ecuador (1.4343°S, 78.3942°W; 3125 m alt.) on 14 August 2018. DHMECN 16608, 16610 and 16613, adult males with the same data as DHMECN 16606. DHMECN 4772, 4777 and 4782 adult females collected by JPRP from Nahuazo-Runtún, Baños, Baños de Agua Santa, Tungurahua, Ecuador (1.4258°S, 78.4257°W; 3100 m alt.) on 28 March 2008 DHMECN 4778, adult male and DHMECN 4779, adult female, collected by JPRP and Nelson Palacios from San Antonio, Baños, Baños de Agua Santa, Tungurahua, Ecuador (1.4572°S, 78.3016°W; 2850 m alt.) on 30 April 2007. DHMECN 4769, adult male collected by JPRP and Salomón Ramírez from Pondoa, Baños, Baños de Agua Santa, Tungurahua, Ecuador (1.4370°S, 78.4426°W; 3000 m alt.) on 31 March 2007. DHMECN 4774 and DHMECN 4781 adult males collected by JPRP from Pondoa, Baños, Baños de Agua Santa, Tungurahua, Ecuador (1.4370°S, -78.4426°W; 3240 m alt.) on 21 March 2007. DHMECN 4807, adult male collected by JPRP, Salomón Ramírez, and Stalin Cáceres, and Luis Recalde from Bosque Protector Cerro Candelaria, Río Verde, Baños de Agua Santa, Tungurahua, Ecuador (1.4572° S, 78.3016°W; 2850 m alt.) on 11 May 2008. DHMECN 18159 and 18160, adult females collected by JPRP, Patricio Vinueza, Paulete Benavidez, and Nantar Kuja in Bosque Protector Guamag, Ulba, Baños de Agua Santa, Tungurahua, Ecuador (1.4194°S, 78.3648°W; 2800 m alt.) on 28 May 2022. DHMECN 18185, adult male collected by JPRP, Patricio Vinueza and Eduardo Peña in Finca Palmonte, Río Negro, Baños de Agua Santa, Tungurahua, Ecuador (1.4349°S, 78.2629°W; 2485 m alt.) on 14 May 2022.

###### Generic placement.

As defined by Lynch and Duellman (1997), Hedges et al. (2008) and Franco-Mena et al. (2023), the *Pristimantismyersi* group (subgenus Trachyphrynus) contains species with the following combination of traits: (1) small body size (SVL in females < 34.6 mm; in males < 20.5 mm); (2) short snout; (3) robust body; (4) Toe V longer than Toe III, Finger I shorter than II; (5) digital discs narrow or slightly expanded (expanded in *P.floridus*); and (6) cranial crests absent. In addition, all species in the group are found on low vegetation or at ground level or even underground. The morphology of the new species agrees with all the aforementioned diagnostic traits; therefore, we place it in the genus *Pristimantis*, subgenus Trachyphrynus.

###### Diagnosis.

**Figure 8.**
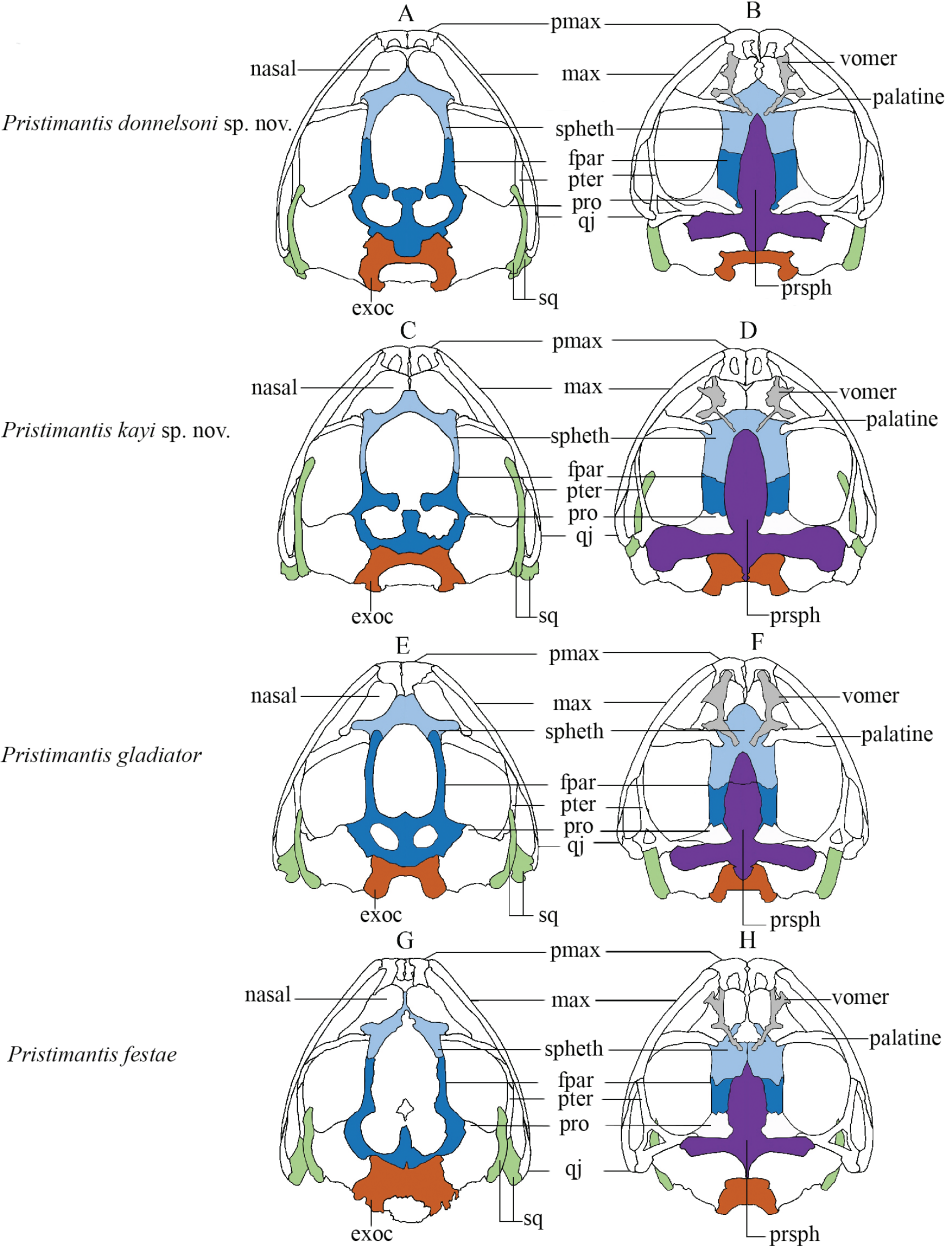
Skull morphology diagram in dorsal (left column) and ventral (right column) views in four species of the *Pristimantismyersi* species group: **A, B***P.donnelsoni* sp. nov., DHMECN 4808 **C, D***P.kayi* sp. nov., DHMECN 14447 **E, F***P.gladiator*DHMECN 12465 **G, H***P.festae*DHMECN 9294. Detail of the skull bones abbreviations: pmax = premaxilla; max = maxilla; spheth = sphenethmoides; fpar = frontoparietal; pter = pterigoides; pro = prootic; q = quadratojugal; sp = squamosal; exo = exoccipital; prph = parasphenoides. Frontoparietal fontanelle not coloured.

**Figure 9.**
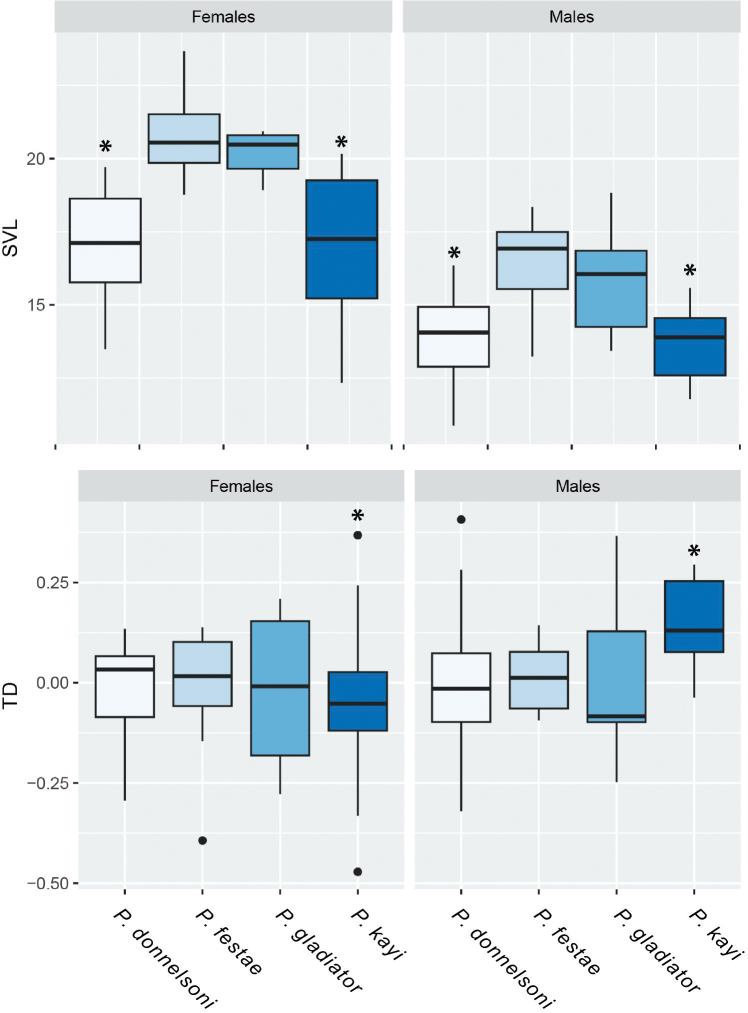
Boxplot of body size (SVL) and tympanic diameter (TD) in four closely-related species of the *Pristimantismyersi* species group. *Asterisks represent species with significant differences.

(1) Skin of dorsum shagreen, occipital fold evident, dorsolateral folds low in banded individuals or weakly defined in uniform or irregular dorsal colour patterns, skin on venter coarsely areolate (Fig. 2); (2) tympanic annulus and tympanic membrane present, tympanum prominent, sexually dimorphic, tympanum in males 7% SVL, tympanum in females 6% SVL; (3) snout subacuminate in dorsal view (tip of snout pointed), rounded in lateral view (Fig. 2); (4) upper eyelid with several small rounded to subconical tubercles; upper eyelid in males 56% IOD (in females 59%) IOD ; cranial crests absent; (5) odontophore processes of the vomer partially concealed, oblique in outline, each processes bearing several small, poorly defined teeth; (6) sphenethmoides with acuminated anterior border dorsally, ventrally present an anteriorly rounded acuminated projected border, posterior border horizontal articulated with frontoparietals; posterior border of frontoparietal present enlarged quadrangular process posteriorly in dorsal view; zygomatic ramus of squamosal short and rounded in dorsal view; procesus cultriform of the parasphenoides reaching posterior level of the vomers, acuminated anterior border (Fig. 8); (7) males with vocal slits and subgular vocal sac; lacking nuptial pads; (8) finger I shorter than II, discs on fingers II–IV rounded (Fig. 3); (9) fingers with slightly visible lateral fringes; (10) ulnar tubercles small, low; (11) heel with small tubercles; outer edge of tarsus with small tubercles, more accentuated in males; (12) inner metatarsal tubercle oval elongated, about twice the size of outer metatarsal tubercle that is rounded; (13) toes lacking lateral fringes, webbing absent; Toe V slightly longer than III, discs slightly expanded (Fig. 3); (14) dorsum brown in several shades, with dark extreme yellow or green tones, females generally darker than males that have usually a banded pattern; banded limbs; venter light brown with dark brown flecks; hidden surfaces of the groin, thighs and armpits pinkish to reddish (in life), chest and throat present light brown tones (Figs 4–7); (15) SVL in males, 14.9–16.4 mm (mean = 13.8, SD = 3.9, n = 32), in females, 13.5–19.7 mm (mean = 17.1, SD = 4.41, n = 18); (16) advertisement call with two notes, duration note of 236–293 ms, with intervals of 522–620 ms; call is comprised by tonal sounds of constant frequency, with a slight upward modulation at end of call; (17) dominant frequency at 2.93 kHz, with two partial harmonics, first one with a range of 5.86–5.94 kHz and the second one with a range of 8.79–8.96 kHz; (18) call duration ranging from 1052–1136 ms.

###### Comparison with other species

**(Figs 5, 8, 9).***Pristimantisdonnelsoni* sp. nov. is smaller than its closer congeners, externally is most similar to *P.gladiator* (Lynch, 1976) and *P.festae* (Peracca, 1904), (Kruskal-Wallis Chi^2^ = 25.39, p = < 0.001, Table 2, Fig. 9), males in *P.gladiator* 15.8 mm vs. *P.donnelsoni* sp. nov. 13.8 mm; females in *P.gladiator* 20.2 vs. *P.donnelsoni* sp. nov. 17.1 mm; males in *P.festae* 16.4 mm vs. *P.donnelsoni* sp. nov. 13.8 mm; females in *P.festae* 20.7 vs. *P.donnelsoni* sp. nov. 17.1 mm. Additionally, the new species differs in having reddish and pinkish marks on the groin and ventral surfaces in several tones of brown, unlike *P.gladiator* which features dark orange marks on a dark brown venter. *Pristimantisdonnelsoni* sp. nov. has a subacuminated snout in comparison to *P.gladiator* which is acuminate. On the other hand, the snout of *P.festae* is rounded in dorsal view and subacuminated in *P.donnelsoni* sp. nov. Another species, closely related and morphologically similar to *P.donnelsoni*, is *P.kayi* sp. nov. Although the two new species are extremely similar, differences in the tympanum diameter are evident, TD in *P.donnelsoni* sp. nov. 0.9 mm vs. *P.kayi* sp. nov. 1.0 mm (Kruskal-Wallis Chi^2^ = 10.28, p = < 0.05*, Table 2, Fig. 8.). *Pristimantisleoni* and *P.sirnigeli* are found at the Western Andean Cordillera, but they lack distinctive concealed reddish flash marks on the groin that are characteristic of *P.donnelsoni* sp. nov.

**Table 2.** Measurements of four closely-related species of *Pristimantis* from the eastern Ecuadorian Andes.

	*Pristimantisdonnelsoni* sp. nov.		*Pristimantiskayi* sp. nov.		*Pristimantisfestae*		*Pristimantisgladiator*	
	Females n = 18	Males n = 32	Females n = 30	Males n = 13	Females n = 10	Males n = 13	Females n = 6	Males n = 14
**SVL**	13.48–19.71 (16.60 ± 4.41)	10.87–16.35 (13.61 ± 3.87)	12.33–20.16 (16.25 ± 5.54)	11.77–15.58 (13.68 ± 2.69)	18.77–23.67 (20.75 ± 1.4)	13.24–18.35 (16.36 ± 1.49)	18.92–20.94 (19.93 ± 1.43)	13.43–18.83 (16.13 ± 3.82)
**HW**	5.42–7.5 (6.46 ± 1.74)	4.3–6.45 (5.38 ± 1.52)	5.16–7.45 (6.31 ± 1.62)	4.37–5.44 (4.91 ± 0.76)	7.09–9.69 (88.22 ± 0.88)	5.04–7.09 (6.44 ± 0.61)	6.85–8.02 (7.44 ± 0.83)	4.97–6.69 (5.83 ± 1.22)
**HL**	5.32–7.54 (6.43 ± 1.57)	4.86–6.58 (5.72 ± 1.22)	5.25–8.11 (6.68 ± 2.02)	4.85–6.28 (5.57 ± 1.01)	6.25–9.34 (7.59 ± 0.89)	5.23–7.54 (6.52 ± 0.62)	7.25–8.21 (7.73 ± 0.68)	5.37–7.11 (6.24 ± 1.23)
**EN**	1.15–1.7 (1.43 ± 0.39)	0.9–1.57 (1.24 ± 0.47)	1.04–2.03 (1.54 ± 0.7)	0.89–1.6 (1.25 ± 0.5)	1.3–2.01 (1.61 ± 0.21)	0.97–1.47 (1.25 ± 0.15)	1.87–2.04 (1.96 ± 0.12)	1.15–1.75 (1.45 ± 0.42)
**IND**	1.39–1.93 (1.66 ± 0.38)	1.13–1.71 (1.42 ± 0.41)	1.32–2.18 (1.75 ± 0.61)	1.27–1.82 (1.55 ± 0.39)	1.75–2.2 (1.98 ± 0.16)	1.4–2.05 (1.73 ± 0.19)	2.06–2.27 (2.17 ± 0.15)	1.52–2.03 (1.78 ± 0.36)
**IOD**	1.66–2.69 (2.18 ± 0.73)	1.61–2.4 (2.01 ± 0.56)	1.67–2.97 (2.32 ± 0.92)	1.54–2.08 (1.81 ± 0.38)	2.23–2.89 (2.58 ± 0.23)	1.85–2.51 (2.19 ± 0.2)	2.3–2.84 (2.57 ± 0.38)	1.71–2.54 (2.13 ± 0.59)
**EW**	1–1.58 (1.29 ± 0.41)	0.8–1.45 (1.13 ± 0.46)	0.85–1.91 (1.38 ± 0.75)	0.76–1.51 (1.14 ± 0.53)	1.13–1.77 (1.51 ± 0.2)	1.05–1.57 (1.31 ± 0.15)	1.22–1.77 (1.5 ± 0.39)	1.01–1.66 (1.34 ± 0.46)
**TD**	0.72–1.17 (0.95 ± 0.32)	0.58–1.34 (0.96 ± 0.54)	0.47–1.49 (0.98 ± 0.72)	0.82–1.19 (1.01 ± 0.36)	0.6–1.23 (0.95 ± 0.18)	0.51–0.96 (0.77 ± 0.12)	1.08–1.6 (1.34 ± 0.37)	0.72–1.59 (1.16 ± 0.62)
**ED**	1.68–2.55 (2.12 ± 0.62)	1.41–1.97 (1.69 ± 0.4)	1.65–2.62 (2.14 ± 0.69)	1.38–2.09 (1.74 ± 0.5)	1.95–2.65 (2.26 ± 0.26)	1.58–2.15 (1.85 ± 0.17)	2.05–2.4 (2.23 ± 0.25)	1.65–2.24 (1.95 ± 0.42)
**TL**	6.19–7.98 (7.09 ± 1.27)	4.95–7.74 (6.35 ± 1.97)	5.89–9.98 (7.94 ± 2.89)	5.32–7.23 (6.28 ± 1.35)	6.89–8.61 (7.76 ± 0.56)	5.56–7.32 (6.42 ± 0.51)	9.63–10.54 (10.09 ± 0.64)	6.88–9.21 (8.05 ± 1.65)
**HaL**	3.09–4.55 (3.82 ± 1.03)	2.71–4.4 (3.56 ± 1.2)	3–5.2 (4.1 ± 1.56)	2.73–3.74 (3.24 ± 0.71)	3.89–5.44 (4.76 ± 0.46)	3.38–4.26 (3.85 ± 0.29)	5.09–5.7 (5.4 ± 0.43)	3.34–4.63 (3.99 ± 0.91)
**FL**	5.62–8.31 (6.97 ± 1.9)	4.72–7.58 (6.15 ± 1.02)	4.9–8.96 (6.93 ± 2.87)	4.69–6.32 (5.51 ± 1.15)	7.1–9.5 (8.28 ± 0.75)	5.87–7.35 (6.63 ± 0.45)	9.21–9.94 (9.58 ± 0.52)	5.78–8.62 (7.2 ± 2.01)
**FW**	0.37–0.64 (0.51 ± 0.19)	0.33–0.63 (0.48 ± 0.21)	0.33–0.89 (0.61 ± 0.4)	0.33–0.55 (0.44 ± 0.16)	0.32–0.61 (0.53 ± 0.09)	0.41–0.51 (0.43 ± 0.03)	0.73–1.04 (0.89 ± 0.22)	0.53–0.79 (0.66 ± 0.18)
**TW**	0.39–0.7 (0.55 ± 0.22)	0.3–0.6 (0.45 ± 0.21)	0.38–0.95 (0.67 ± 0.4)	0.38–0.65 (0.52 ± 0.19)	0.3–0.64 (0.51 ± 0.09)	0.38–0.51 (0.46 ± 0.04)	0.72–1.09 (0.91 ± 0.26)	0.41–0.81 (0.61 ± 0.28)

Legend: SVL (snout–vent length), HW (head width), HL (head length), EN (eye–nostril distance), IND (internarial distance), IOD (interorbital distance), EW (upper eyelid width), TD (tympanum diameter) eye–nostril, ED (eye diameter), TL (tibia length), HaL (Hand length), FL (foot length), FW (Finger III width), TW (Toe IV length).

Differences in the skull morphology amongst closely-related species are summarised in Table 3 as follows: *Pristimantisdonnelsoni* sp. nov. has the posterior border of the frontoparietal with an elongated quadrangular crested process projected posteriorly; while in *P.gladiator*, it is irregular and slightly projected posteriorly; in *P.kayi* sp. nov. posterior border of frontoparietal, it is irregular and not projected; finally, *P.festae* exhibit frontoparietal with rounded posterior border not projected.

**Tablе 3.** Skull diagnostic characters between four related species of *Pristimantisgladiator* complex.

Species	Sphenethmoides in ventral view	Frontoparietals	Squamosal	Parasphenoides
*Pristimantisdonnelsoni* sp. nov.	The anterior border is triangular and the posterior border presents horizontal articulations with frontoparietals	Posterior border with elongated quadrangular crested process posteriorly	Short and rounded zygomatic ramus	The cultriform process reaches the posterior level of vomers and ends in a sharp anterior border; does not reach the posterior border of the skull, lateral ala of parasphenoid short does not reach the level of the maxilla
*P.kayi* sp. nov.	The anterior border is truncated or blunt and not projected and the posterior present oblique articulation with frontoparietals	The posterior border is irregular and not projected	Elongated and sharp zygomatic ramus, reaching maxillae	The cultriform process reaches the posterior level of vomers and ends in a blunt anterior border
*P.gladiator*	The anterior border is rounded and projected anteriorly, posterior border presents a pair of suboval processes posterior, articulated with frontoparietals	The posterior border is irregular and slightly projected posteriorly	Short and blunt zygomatic ramus	The cultriform process does not reach the posterior region of vomers with a subacuminated anterior border
*P.festae*	The anterior border is not projected and the posterior is serrated	Round posterior border not projected	Very short and blunt zygomatic ramus not extending to the maxillae	The cultriform process is short and does not reach vomers, present and anterior short blunt border and posterior serrated border its anterior border is short and very acute

The length and shape of the zygomatic ramus in the squamosal, are short and rounded in *Pristimantisdonnelsoni* sp. nov.; *P.gladiator* presents a short and blunt zygomatic ramus; *P.kayi* sp. nov. shows an elongated and sharp zygomatic ramus, reaching level of the maxillae; whereas in *P.festae*, zygomatic ramus is very short and blunt, not extending to the level of the maxillae (Fig. 8).

In ventral view of the skull, differences are evident in *P.donnelsoni* sp. nov., the anterior border of sphenethmoides is triangular and posterior border presents horizontal articulations with frontoparietals; in *P.gladiator*, anterior border of sphenethmoides is rounded and projected anteriorly, posterior border presents a pair of posterior suboval processes, articulated with frontoparietals; in *P.kayi* sp. nov., the anterior border of sphenethmoides is blunt and not projected and the posterior border presents oblique articulation with frontoparietals.

In *P.donnelsoni* sp. nov., the parasphenoid does not reach posterior border of the skull, lateral alary process of parasphenoid is short and does not reach level of maxilla, the cultriform process of the parasphenoid reaches posterior level of vomers and ends in a sharp anterior border, differing from *P.gladiator*, in which parasphenoid does not reach the posterior region of vomers with a subacuminated anterior border; in *P.kayi* sp. nov., the cultriform process of the parasphenoid reaches the posterior level of vomers and ends in a blunt anterior border, in *P.festae*, the sphenethmoides presents an anterior border of cultriform process and does not reach vomers, its anterior border is short and very acute (Table 3, Fig. 8).

###### Description of the holotype

**(Figs 2, 3, 4).** Adult female (DHMECN 14701) with robust body and short limbs; head slightly longer than wide, not as wide as body, head width 40% of SVL (15.4 mm); head length 41% of SVL; snout short, with a fleshly terminal rounded tip, subacuminated in dorsal view and rounded in lateral view; eye–nostril distance 9.9% of SVL; nostrils narrow higher than long, laterally directed; canthus rostralis angular in dorsal and lateral view; loreal region slightly concave; lips rounded; upper eyelid bearing small low rounded tubercles; snout and interorbital region with scattered small low rounded tubercles; upper eyelid width 49.3% of IOD; tympanic annulus visible on its 3⁄4 part, with the dorsal margins obscured by supratympanic fold; tympanic membrane present, distinct; tympanum diameter 43.6% of eye diameter, one subconic postrictal tubercle surrounded by little low tubercles; rounded tubercles along the supratympanic fold and 2–3 rounded tubercles surrounded by small tubercles on the temporal region; Choanae small, rounded, not concealed by palatal shelf of maxilla; dentigerous processes of vomers low, distinct, oblique in outline, widely separated, positioned posteromedial to choanae; each vomer bearing 2 or 3 teeth; tongue twice as long as wide, free posteriorly on two-thirds of its length. Temporal folds are formed by a row of small low tubercles, extending from the posterior margin of the upper eyelid to the suprascapular region. Skin on the dorsum with scattered, low-rounded tubercles, small folds formed by little rounded tubercles on upper areas of flanks; lower flanks finely granular; skin on the upper surfaces of forelimbs and hind limbs with indistinct rows of low rounded tubercles.

Skin of the throat, chest, and ventral surfaces and limbs is weakly areolate, venter coarsely areolate; discoidal fold weakly defined; cloacal sheath short; skin in the cloacal region smooth. Row of 2 or 3 rounded ulnar tubercles; palmar tubercles low, outer palmar tubercle bifid, V-shaped, approximately twice as large as elongate ovoid thenar tubercle; subarticular tubercles low, well-defined, rounded in ventral view and flattened in lateral view; supernumerary tubercle at the base of fingers present, indistinct; fingers bearing lateral fringes; Finger I shorter than Finger II; discs on Fingers I and II, rounded and slightly expanded; discs more expanded on Fingers III and IV; ventral pads on fingers well-defined by circumferential grooves on Fingers II, III and IV, not evident on Finger I (Fig. 3).

Hind limbs robust, tibia length 47.54% of SVL; foot length 47.67% of SVL; upper surfaces of hind limbs with low rounded tubercles; heel bearing one to four low rounded tubercles; the outer surface of tarsus bearing a row of four low rounded tubercles; inner tarsal fold present; inner metatarsal tubercle evident, oval, elongate elliptical, three times size of outer metatarsal tubercle; inner plantar surface smooth; subarticular tubercles poorly defined, rounded in ventral view and flattened in lateral view; toes lacking lateral fringes; webbing between toes absent; discs on toes rounded, slightly expanded; all toes with ventral pads defined by circumferential grooves, less distinct on Toe I; relative lengths of toes: I < II < III < V < IV (Fig. 3); Toe V slightly longer than III, disc of Toe III not reaching medial subarticular tubercle of Toe IV; the distal end of the disc on Toe V reaches the medial subarticular tubercle of Toe IV.

###### Measurements of the holotype

**(in mm).** Adult female, DHMECN 14701, SVL = 15.4; Tibia Length = 7.3; Foot Length = 7.4; Hand Length = 3.8; Head Length = 6.3; Head Width = 6.2; Eye Diameter = 1.9; Tympanum Diameter = 0.8; Forearm Length = 4.2; Snout Length = 5.3; Tarsus Length = 4.2; Thigh Length = 5.5; Upper Arm Length = 3.4; Interorbital Distance = 2.1; upper Eyelid Width = 1.0; Internarial Distance = 1.5; Eye–Nostril distance = 1.5; Wide Finger III = 0.4; Toe IV Width = 0.4.

###### Colour of holotype in life

**(Figs 4, 5, 7).** Head, sides of the head, dorsum, flanks and limbs brown, with a large rounded white blotch on the middle of dorsum; light and dark brown banded lips, the broad dark brown supratympanic band extends from the tympanum to level of insertion of arm; flanks darker than dorsum with oblique, pale and diffuse bands; several pinkish-whitish markings scattered near the groin and axilla; limbs banded in light and dark brown tones. Throat and venter dark brown covered with scattered tiny white spots; ventral surfaces of limbs lighter brown with grey tones. Iris silver with a dark copper-coloured horizontal bar.

###### Colour of holotype in ethanol

**(Figs 2, 3, 6).** Dorsum dark brown with a mid-dorsal white spot; limbs banded with light brown; lips banded; dark supratympanic band delineated below with thin pale line; flanks dark brown to black with pale oblique bands; several white markings near groin and axilla; chin with brown marking outlined with pale shades; throat brown with pale spots; belly dark brown covered with minute white spots; ventral surfaces of limbs and palms light brown with pale spots.

###### Osteology of the skull.

The skull of the adult female paratype DHMECN 4808, is illustrated on its dorsal and ventral surfaces in Fig. 8. We describe main skull bones with diagnostic characters and the key differences are summarised in Table 3. Skull is slightly longer than wide. Dorsally paired nasals overlap the sphenethmoid, the sphenethmoid articulates posteriorly with the frontoparietal, posterior border of frontoparietal present quadrangular projected process posteriorly. A large frontoparietal fontanelle, connected to two parietal fontanelles, is delimited by the sphenethmoid and the frontoparietals.

In ventral view, anterior margin of the sphenethmoid is acuminated. Palatines overlap with the sphenethmoid and vomers. The vomer is narrowly separated medially; each vomer has three distinctive rami dentigerous process of the vomer reaches the level of the palatine. The parasphenoid present an inverted cross shape; cultriform process with an acute anterior border that reaches level of vomers, the alary processes are directed transversely, partially covering the otic area; the posterior process of the parasphenoid does not reach the foramen magnum. In dorsal view, zygomatic ramus of squamosal presents a short and rounded anterior border extending backwards to the level of the maxilla (Fig. 8).

###### Variation

**(Figs 5, 6, 7, 9).***Pristimantisdonnelsoni* is highly polymorphic and sexually dimorphic species (Suppl. material 1). Females show dark brown dorsal colouration with black to brown banded flanks and tiny white spots; brown with white spots on dorsum; white interorbital line from snout to cloaca, whitish interorbital band, broad brown dorsal mid-dorsal band delineated towards flanks with dark brown to yellowish to black. Some individuals with shades of green. Venter brown in various shades with small whitish spots; some individuals dark brown-grey with a cross pattern along the mid-line and shoulder girdle, white line delineating the outer edge of the tarsus, leg and cloaca. Males are smaller than females and characterised by dorsal banding patterns in shades of grey, brown, cream and yellowish, some individuals with pale dorsolateral lines along the dorsolateral folds; white interorbital bar; hidden groin surfaces and armpits with white or pale markings mottled with shades of grey or brown. Some individuals with shades of green on the dorsum and flanks.

###### Call description

**Figure 10.**
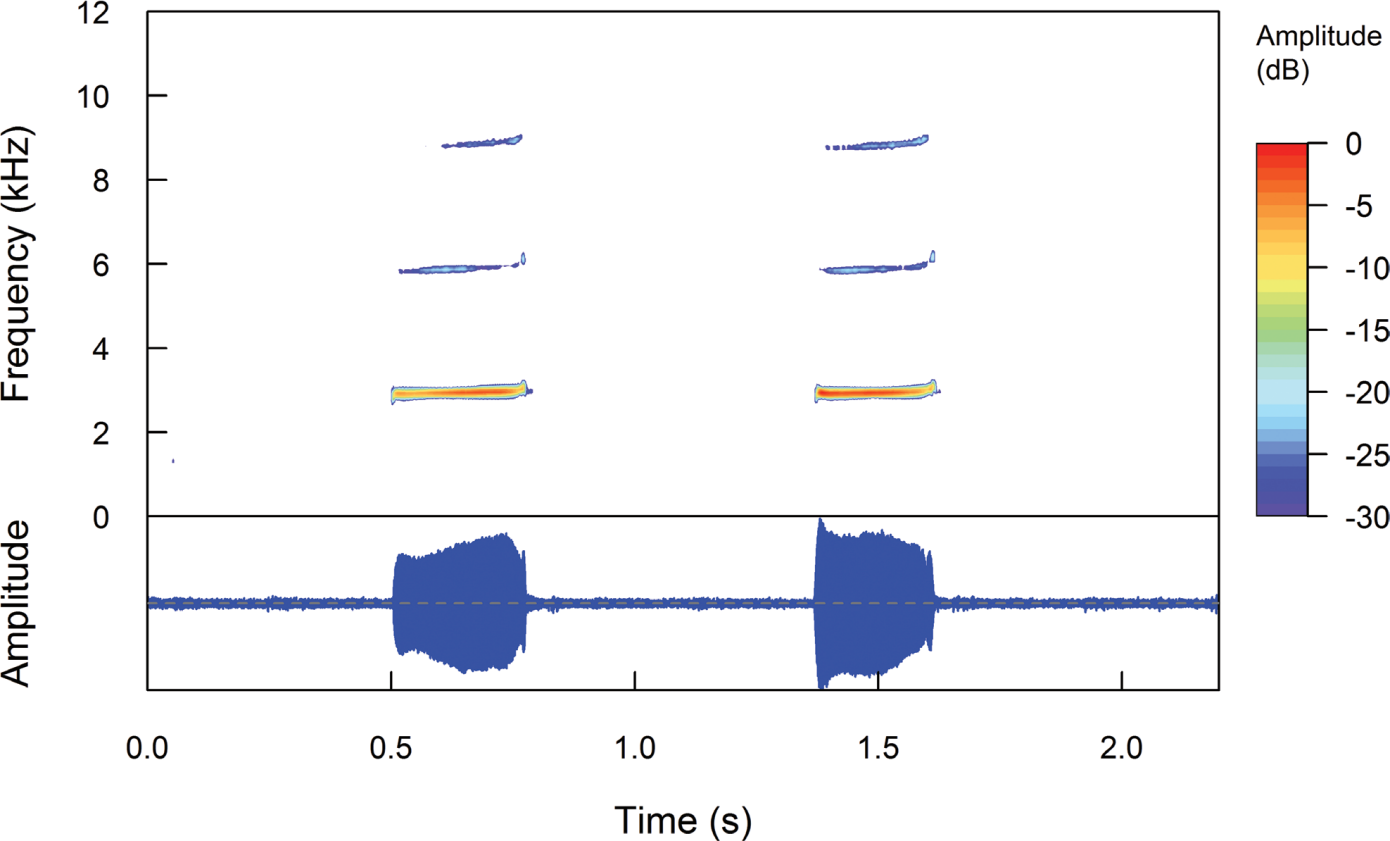
Oscillogram and spectrogram of the advertisement call of *Pristimantisdonnelsoni* sp. nov., adult male, paratype DHMECN 4774.

**(Fig. 10).** The call description of *Pristimantisdonnelsoni* sp. nov. is based on recordings of a male paratype DHMECN 4774, on 21 March 2007, 19:00 h, made by JPRP, on the northern flank of Tungurahua Volcano, no environmental parameters were obtained. The calling male was sitting on herbaceous plants on the forest floor. The call is comprised by tonal sounds of constant frequency, with a slight upward modulation at the end of the song. It presents a dominant frequency at 2.93 kHz, with two partial harmonics, first one with a range of 5.86–5.94 kHz and the second one with a range of 8.79–8.96 kHz. It has a duration ranging from 1052–1136 ms, with intervals ranging from 4531–5985 ms, emitting at a rate ranging from 8.43–10.60 calls/minute. The calls are composed of two notes. The notes have a duration ranging from 236–293 ms, with intervals ranging from 522–620 ms.

###### Distribution and natural history observations.

**Figure 11.**
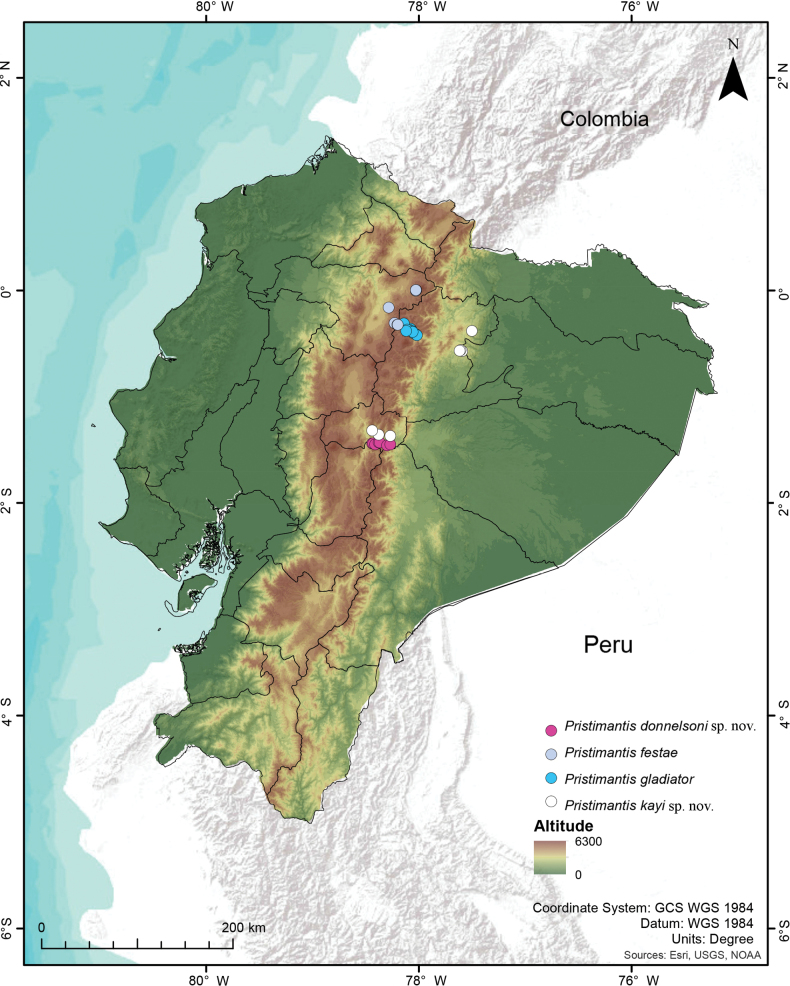
Distribution of four closely-related species of the *Pristimantismyersi* species group in the Andes of Ecuador.

**Figure 12.**
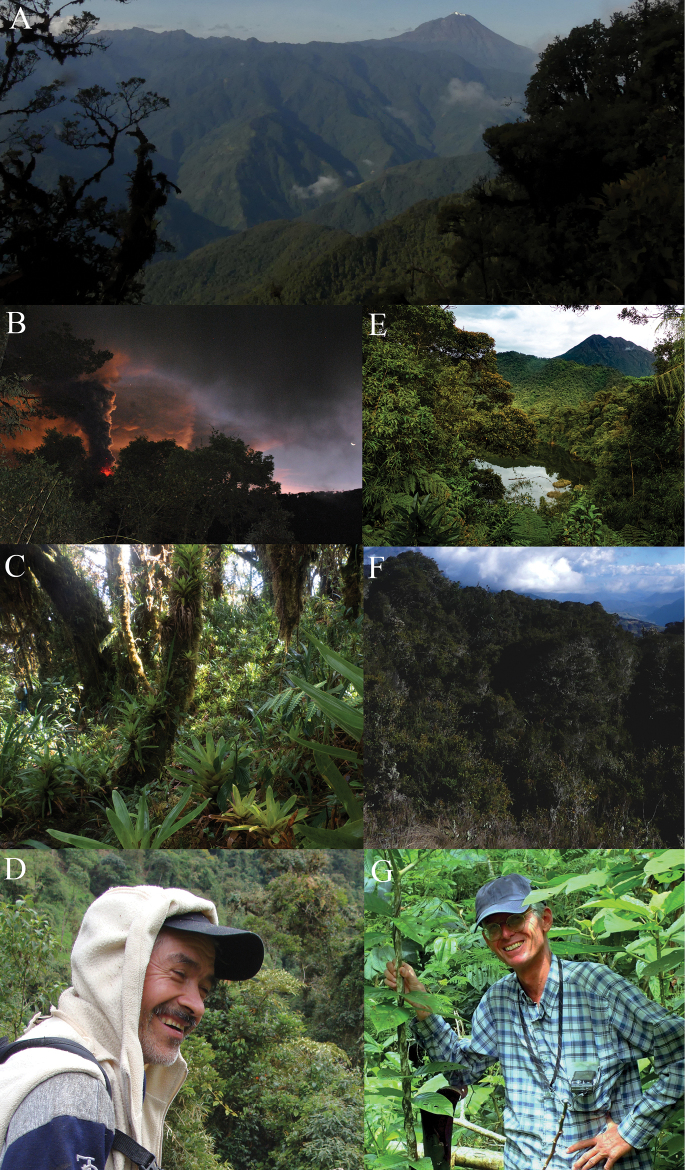
Habitat vegetation at type localities and etymology of the two new frogs described herein. Photographs by JPRP and Zane Libke **A** panoramic view of the upper Pastaza Valley and its adjacent mountains, main geographic barriers promoting speciation at a regional scale, at back Tungurahua Volcano and mountains south of the Pastaza River habitat of *Pristimantisdonnelsoni* sp. nov., image from the slopes of Cerro Mayordomo type locality of *Pristimantiskayi* sp. nov., in the northern mountains to the Pastaza River **B** Tungurahua Volcano eruption, from Chamana Reserve **C** Montane forest in Candelaria Reserve **D** Don Nelson Palacios (+) during an expedition; we tribute his work with the naming of *Pristimantisdonnelsoni* sp. nov. **E** Sumaco Volcano seen from Wawa Sumaku, habitat of *P.kayi* sp. nov. **F** Montane forest and subpáramo in Leito, Patate **G** Andreas Kay (+); we honour his devotion to nature photography and research in the upper Pastaza Valley and through Ecuador by naming *Pristimantiskayi* sp. nov.

*Pristimantisdonnelsoni* sp. nov. is known from seven localities on the southern mountains of the Pastaza drainage, Tungurahua Province, Ecuador (Fig. 11). Three localities are located on the Tungurahua Volcano, whereas the others correspond to nearby protected areas (Chamana Reserve and Cerro Candelaria Reserve, Finca Palmonte and Bosque Protector Hacienda Guamag), all at elevations of 2950–3800 m. The collection localities range from high Andean montane forest, with trees of the genera *Ceroxylum*, *Clusia*, *Weinmannia*, and others, between 10–20 m high, with abundant epiphytes, bromeliads orchids, and mosses, to paramo habitat which is mainly grassland with shrubs in the higher part (Fig. 12). Most of the individuals were found sitting on herbs or on leaf litter on the forest floor, with dense ferns and small herbaceous plants, always near ground level (< 30 cm above ground). Calling males are active during the day and sporadically call at night. Sympatric species include Pristimantisaff.devillei, *P.modipeplus* in Tungurahua Volcano, *P.puruscafeum* and. *P.marcoreyesi* and *P.lacrimosus* group sp. nov., in Cerro Candelaria and Chamana.

###### Etymology.

The specific epithet “donnelsoni” is a noun in the genitive case and a patronym for Don Nelson Palacios (Fig. 12D). We named this new species after him as a tribute to his friendship and collaboration in the first collections of new species described during the last decade and several additional important localities within the upper basin of the Pastaza River. This is a special recognition to him for eternity.

##### ﻿ Pristimantis kayi

Taxon classificationAnimaliaAnuraStrabomantidae

Juan M. Guayasamin, Miguel Urgilés-Merchán, Daniela Franco-Mena, Carolina Reyes-Puig, Diego Batallas & Juan Pablo Reyes-Puig sp. nov.

10218311-9884-57C8-AE06-8B321C63C959

https://zoobank.org/E4854476-B0BD-44DB-8E5F-3D4E2A590962

Figs 1 , 5 , 8 , 12 , 13 , 14 , 15 , 16 , 17

###### Type material.

**Figure 13.**
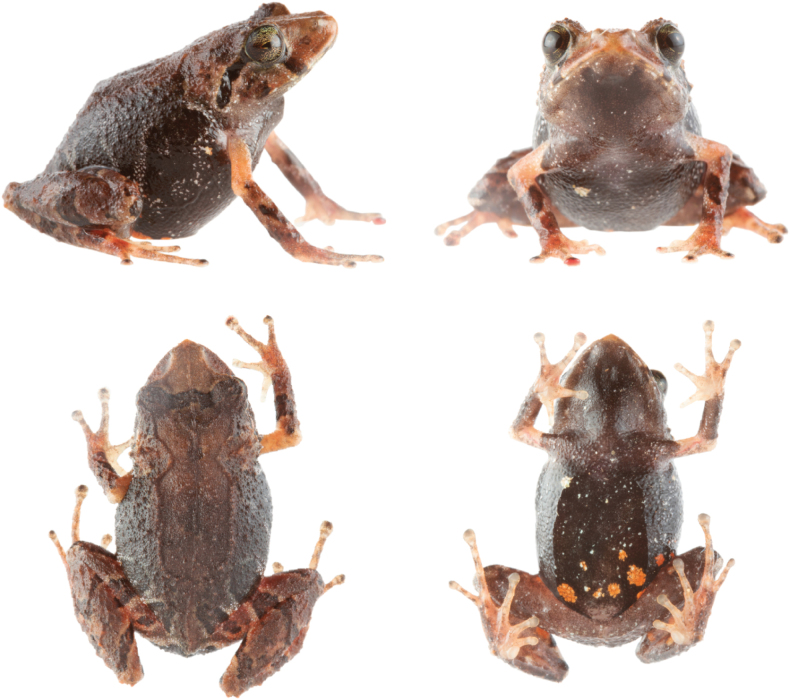
Holotype of *Pristimantiskayi* sp. nov. in life, adult female, ZSFQ 0775. LRC: 19.7 mm. Photographs by Jose Vieira.

***Holotype*.**ZSFQ 0775, adult female (Figs 5C, 13, 15–17) collected by José Vieira, David Brito-Zapata, Jefferson Mora, and Carolina Meneses from Volcán Sumaco, Sumaco National Park, Napo, Ecuador (0.5696°S, 77.5939°W; 2322 m alt.) on 11 September 2018.

**Figure 14.**
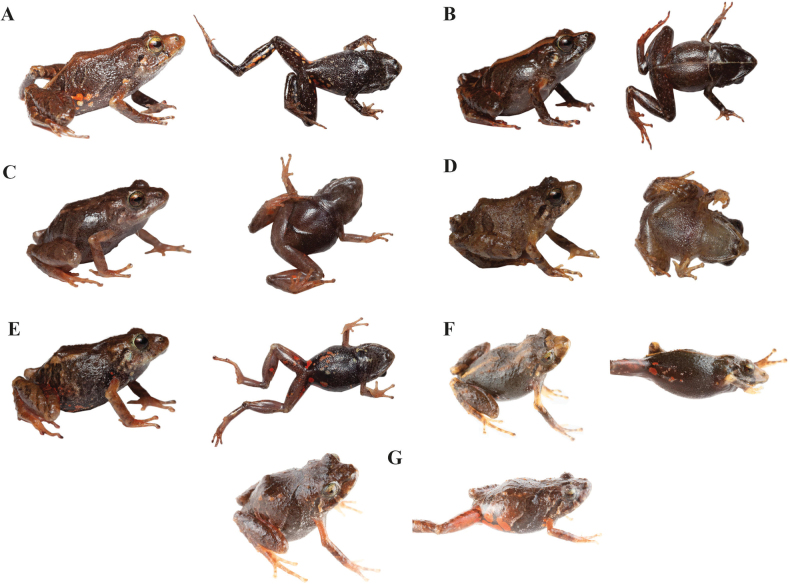
Colour variation of *Pristimantiskayi* sp. nov. **A**MZUTI 2209, adult female, LRC: 20.15 mm, from Cordillera de Guacamayos **B**MZUTI 2210, adult female, LRC:19.90 mm, from Cordillera de Guacamayos **C**MZUTI 2211, adult male, LRC: 14.30 mm, from Cordillera de Guacamayos **D**MZUTI 2213, adult male, LRC: 15.10 mm, from Cordillera de Guacamayos **E**MZUTI 2214, adult female, LRC: 18.73 mm, from Cordillera de Guacamayos **F**ZSFQ 774, adult female, LRC:18.96 mm from Sumaco Volcano **G**ZSFQ 782, adult female, LRC: 30.03 mm, from Sumaco Volcano. Photographs by Lucas Bustamante (**A, B, C, D, E**) and Jose Vieira (**F, G**).

***Paratypes*** (13, 4 ♂ / 9 ♀, Figs 14, 17). ZSFQ 0779, adult female. ZSFQ 773, adult male. ZSFQ 782, adult female with the same data as the holotype, ZSFQ 783, adult female collected by José Vieira, David Brito-Zapata, Jefferson Mora, and Carolina Meneses from Sumaco National Park, Napo, Ecuador (0.5696°S, 77.5753°W; 2,476 m alt.) on 1 September 2018; ZSFQ 778, adult male, ZSFQ 780 and 781 with same data as ZSFQ 783. DHMECN 14446, adult female collected by Mario Humberto Yánez Muñoz, JPRP, and DFM from Cerro Mayordomo, Reserva Machay, Río Negro, Baños de Agua Santa, Tungurahua, Ecuador (1.3686°S, 78.2693°W 8; 2972 m alt.) on 2 March 2018. DHMECN 15226 and 15237, adult females collected by Kelsey Huisman and Eduardo Peña from Vizcaya, Reserva Naturetrek, Ulba, Baños de Agua Santa, Tungurahua, Ecuador (1.396°S, 78.3942°W; 3150 m alt.) on 12 December 2019. DHMECN 15231 and 15233, adult females collected by Kelsey Huisman and Eduardo Peña from Cerro Mayordomo, Reserva Ecológica Machay, Río Verde, Baños de Agua Santa, Tungurahua, Ecuador (1.368°S, 78.2692°W; 2969 m alt.) on 24 November 2019. DHMECN 16212, adult female collected by JPRP fromVizcaya, Reserva Naturetrek, Ulba, Baños de Agua Santa, Tungurahua, Ecuador (1.3962°S, 78.3941°W; 3062 m alt.) on May 2021. DHMECN 18440, adult male collected by JPRP, Eduardo Peña, and Edgar Martínez, from Los Mortiños, Leito, Patate, Tungurahua, Ecuador (-1.315235, -78.452793; 3448 m alt.) on 6 July 2022. MZUTI 2209, adult female collected by Juan M. Guayasamin and Lucas Bustamante from Cordillera de los Guacamayos, Napo, Ecuador (0.3769°S, 77.5048°W; 2190–2247 m alt.) in March 2013. MZUTI 2010, 2011, 2013 and 2014 with the same data as MZUTI 2209.

###### Generic placement.

As defined by Lynch and Duellman (1997), Hedges et al. (2008), and Franco-Mena et al. (2023), the *Pristimantismyersi* group (subgenus Trachyphrynus) contains frogs with the following combination of traits: (1) small body size (SVL in females < 34.6 mm; in males < 20.5 mm); (2) short snout; (3) robust body; (4) Toe V longer than Toe III, Finger I shorter than II; (5) digital discs narrow or slightly expanded (expanded in *P.floridus*); and (6) cranial crests absent. In addition, all species in the group are found on low vegetation or at ground level, or even underground. The morphology of the new species agrees with all the aforementioned diagnostic traits.

###### Diagnosis.

**Figure 15.**
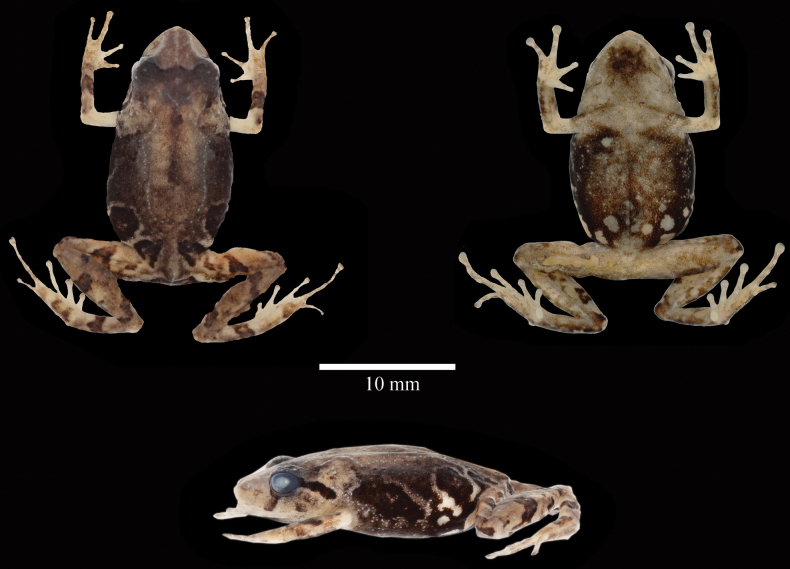
Preserved holotype of *Pristimantiskayi* sp. nov. ZSFQ 0775, on its dorsal, ventral and lateral views. Photographs by David Brito-Zapata.

**Figure 16.**
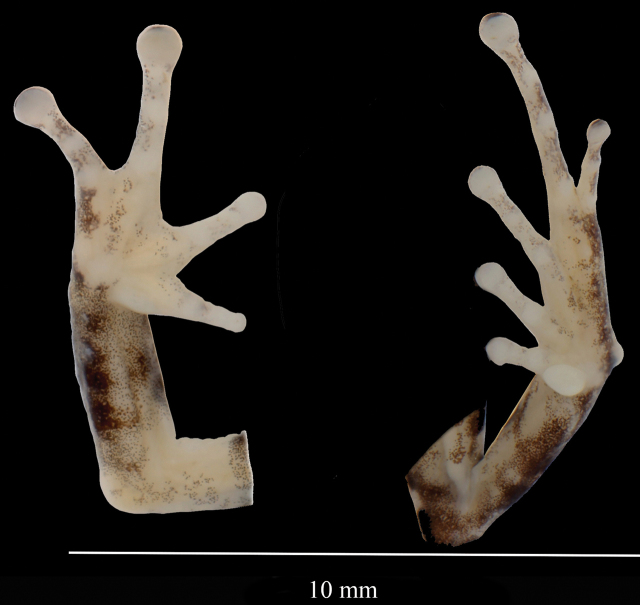
Detailed ventral view of hand and feet of the holotype ZSFQ 0775 of *Pristimantiskayi* sp. nov. Photographs David Brito-Zapata.

**Figure 17.**
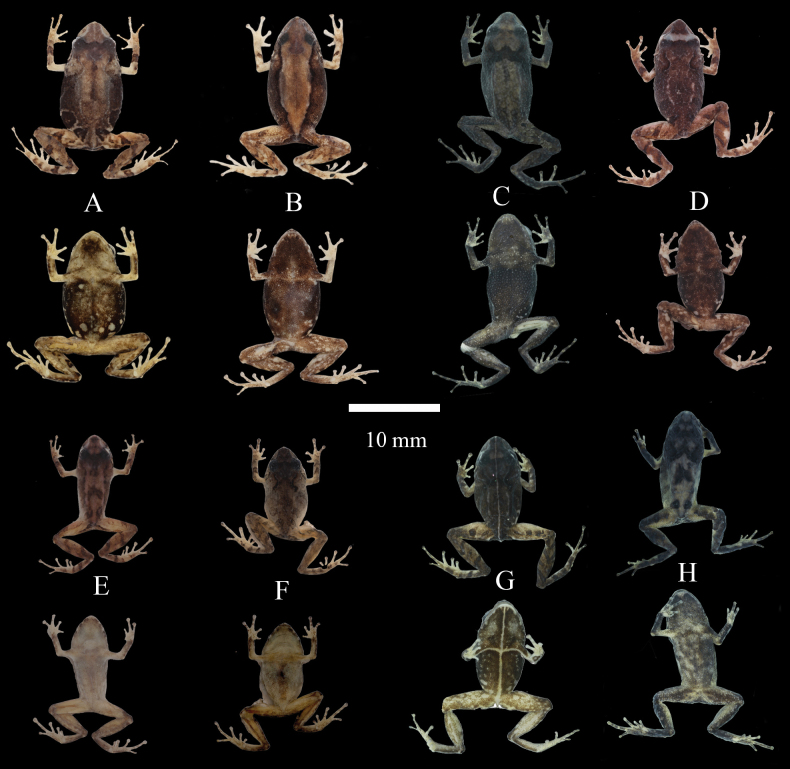
Dorsal and ventral views of preserved type series of *Pristimantiskayi* sp. nov. **A** holotype ZSFQ 0775, female; Paratypes **B**ZSFQ 0779, female **C**DHMECN 16212, female **D**MZUTI 5162, female **E**MZUTI 4657, male **F**ZSFQ 0773, male **G**DHMECN 15233, male **H**DHMECN 18440, male; Photographs by JPRPR and David Brito-Zapata.

(1) Skin on dorsum shagreen with small scattered, rounded warts; upper flanks with numerous low warts; dorsolateral and w-shaped occipital folds present (Figs 13–15); (2) tympanic membrane and tympanic annulus well differentiated (Fig. 12); tympanic annulus not sexually dimorphic; tympanum in males 7% SVL, tympanum in females 7% SVL; (3) snout rounded in dorsal, protruding in lateral view (Fig. 15); (4) upper eyelid usually bearing one conical or subconical tubercle and many low tubercles; upper eyelid about in males 63% IOD, in females 59% IOD ; cranial crests absent; (5) dentigerous processes of the vomer evident, each process bearing 2–3 teeth; (6) sphenethmoides on its dorsal view with short blunt anterior border, is short and blunt in ventral view, posterior border present oblique articulation with frontoparietals; posterior border of frontoparietal with an irregular border not projected in dorsal view; zygomatic ramus of squamosal elongated anteriorly, in dorsal view; procesus cultriform of the parasphenoides reaching posterior level of the vomers, rounded anterior border (Fig. 8); (7) males with vocal slits, nuptial pads absent; (8) finger I shorter than finger II; disc on Finger I not expanded; discs on Finger II–IV slightly expanded (Figs 6, 15); (9) fingers with thin lateral fringes; (10) two or three low ulnar tubercles present; (11) heel with low to conical tubercle; outer edge of tarsus with low conical tubercles; (12) inner metatarsal tubercle oval, about 1.5–2× the length of round outer metatarsal tubercle; (13) toes with thin lateral fringes; webbing absent; Toe V slightly longer than Toe III; discs slightly expanded (Figs 15, 16); (14) in life, dorsum brown with darker and lighter markings; venter light grey to black, with or without orange to red spots of different sizes; groin with orange to red spots that are more conspicuous in females than males (see Colour variation; Figs 14, 17); (15) SVL in adult males, 11.8–15.6 mm (mean = 13.7, SD = 2.7, n = 30), SVL in adult females 12.3–20.2 mm (mean = 16.3, SD = 5.5, n = 13); (16) call composed of 1 to 7 notes, notes have a mean duration of 36.0 ± 18.3 ms, the mean interval between notes is 43.8 ± 36.2 ms, emitted at a mean rate of 15.7 ± 6.1 notes/second. The notes are composed of 2 to 13 pulses. Pulses have a mean duration of 3.9 ± 1.1 ms, with a mean interval between pulses of 4.2 ± 2.8 ms, emitted at a mean rate of 202.2 ± 119.7 pulses/second; (17) dominant frequency of 3.19 ± 0.05 kHz, with 2–6 partial harmonics in the spectrogram; (18) call duration of 165.1 ± 130.1 ms.

###### Comparison with other species

**(Figs 5, 8, 9).***Pristimantiskayi* sp. nov. is most similar to *P.gladiator* (Lynch, 1976), *P.festae* (Peracca, 1904), and *P.donnelsoni* sp. nov. *Pristimantiskayi* sp. nov. differs from *P.gladiator* and *P.festae* by being smaller (Kruskal-Wallis Chi^2^ = 25.39, p = < 0.001, Table 2, Fig. 9). Additionally, *P.kayi* sp. nov. differs from *P.festae* by having orange to red spots on the groin (white to reddish large spots in *P.festae*; Fig. 5) and slightly expanded discs on outer fingers (discs not expanded in *P.festae*). *Pristimantiskayi* sp. nov. is similar to the allopatric *P.donnelsoni* sp. nov.; however, the diameter of the tympanum in males is larger in *P.kayi* sp. nov. compared to *P.donnelsoni* sp. nov. and the other closely-related species (i.e. *P.festae* and *P.gladiator*) (Kruskal-Wallis Chi^2^ = 10.28, p = < 0.05*, Table 2, Fig. 9). The most conspicuous differences in the skull morphology between the two new species are listed below (Fig. 8): in *P.kayi* sp. nov., the parasphenoid is much more developed than in *P.donnelsoni* sp. nov., with its lateral alary processes almost reaching the squamosal; also, the palatine bones are shorter in *P.kayi* sp. nov. than in *P.donnelsoni* sp. nov. Finally, the zygomatic ramus of the squamosal is much longer in *P.kayi* sp. nov. than in *P.donnelsoni* sp. nov. (Fig. 8).

###### Description of the holotype

**(Figs 13, 15, 16).** Adult female (ZSFQ 0775) robust body; head slightly longer than wide, not as wide as body, head width 35.27% of SVL (19.7); head length 38% of SVL; snout rounded in dorsal view, protruding in lateral view; eye-nostril distance 9% of SVL; with small papilla at tip (Fig. 1); in lateral view, distinct rostral ridge; loreal region slightly concave; nostrils protruding, laterally directed; interorbital area flat, wider than upper eyelid (upper eyelid width 62% of OID); cranial ridges absent; upper eyelid with one conical and several non-conical tubercles; tympanic membrane well defined, pigmented like surrounding skin; tympanic ring distinct, round; supratympanic fold present, obscuring anterodorsal and posterodorsal edges of the ring; tympanic diameter 46% of eye length; several low to conical tubercles situated in the area just posterior to tympanum. Choanae are small, with an oval to square shape, not concealed by the palatal shelf of maxillary; the dentigerous process of the vomer oblique, widely separated, posteromedial to choanae, each bearing two small teeth; tongue slightly longer than wide, granular, with a conspicuous notch along the posterior border.

The skin of the head is greyish; the dorsum greyish, with scattered small tubercles, some of which are aligned over a W-shaped occipital marking, forming folds; upper flanks with numerous low warts; venter slightly areolate; discoidal fold absent; the cloacal sheath is absent; fingers with thin lateral bangs; length of fingers I < II < IV < III; palmar tubercle round, thenar tubercle oval (Fig. 16); subarticular tubercles round, not prominent; supernumerary palmar tubercles not evident; disc sheath of Finger I not expanded; those of Fingers II–IV slightly expanded; all disc sheaths with nearly elliptical ventral pads defined by grooves (Fig. 16).

Hind limbs relatively robust; tibia length 47% SVL; foot length slightly smaller than tibia length (foot length 44% SVL); tarsal tubercles present; small, conical tubercle on heel; toes with narrow lateral fringes (Fig. 16); subarticular tubercles round, not prominent; inner metatarsal tubercle oval, two times the size of outer tubercle; few low supernumerary plantar tubercles present (Fig. 16); disc covers of Toes slightly expanded; toes with defined pads; disc pads nearly elliptical; toe lengths I < II < III < V < IV (Fig. 16); the tip of Toe V reaches the middle level of penultimate subarticular tubercle of Toe IV; the tip of Toe III almost reaches the proximal border of penultimate subarticular tubercle of Toe IV.

###### Measurements of the holotype

**(in mm).** Adult female, ZSFQ 0775. SVL = 19.7; Tibia Length = 9.3; Foot Length = 8.6; Hand Length = 5.0; Head Length = 7.5; Head Width = 7.0; Eye Diameter = 2.4; Tympanum Diameter = 1.1; forearm length = 4.4; snout length = 5.6; Tarsus length = 5.3; Thigh Length = 8.3; Upper arm Length = 3.1; Interorbital Distance = 2.6; Upper Eyelid Width = 1.6; Internarial Distance = 2.1; Eye–Nostril distance = 1.8; Finger III Width = 0.8; Toe IV Width = 0.3.

###### Colour of holotype in life

**(Fig. 13).** Head, sides of the head, dorsum, flanks and limbs brown, with darker interorbital bar, lips banded with light and dark brown; broad dark brown supratympanic band extending from region anterior to tympanum to before arm insertion. Flanks lighter than dorsum with oblique, dark bands, several scattered whitish markings near the groin and a white spot in the axilla; limbs banded in shades of dark brown. The throat and belly are dark brown and covered with scattered small yellowish-white spots; the lower ventral part has orange spots; the ventral surfaces of the forelimbs are lighter brown. Iris golden with dark brown reticulations, with a reddish-copper horizontal bar.

###### Colouration of holotype in ethanol

**(Figs 15, 16).** The holotype has the following colour pattern: head pale brown with darker interorbital and labial bars; black supratympanic stripe; dorsum brown with several darker markings. Flanks grey to black with minute white spots and lighter diagonal stripes. Groin black with large white spots. The dorsal surfaces of limbs are pale brown with dark brown bars. Cloacal region pale brown, delimited by supracloacal black stripe. The anterior and posterior surfaces of the thighs are brown with several medium to large cream spots. Throat and chest cream-brown with darker marks on the centre (Fig. 15). Venter dark grey, with minute to large white spots. Palms and soles are mostly cream, with some pigmentation of Finger IV and Toe V (Fig. 16).

###### Osteology of the skull.

The skull of the adult female paratype DHMECN 14447 is illustrated on its dorsal and ventral surfaces in Fig. 8. We describe the main skull bones with diagnostic characters in Table 3. Skull is slightly longer than wide. Dorsally paired nasals overlap the sphenethmoid; the sphenethmoid articulates posteriorly with the frontoparietals, posterior border of frontoparietal is irregular and not projected. A large frontoparietal fontanelle, connected to two parietal fontanelles, is delimited by the sphenethmoid and the frontoparietals.

In ventral view, anterior margin of the sphenethmoid is rounded. The palatines are relatively short and overlap the sphenethmoid on its anterior portion. Vomers are narrowly separated medially; each vomer has three distinctive rami; the dentigerous process of the vomer does not reach the level of the palatine and almost contacts the tip of the cultriform process of the parasphenoid. The large parasphenoid presents the shape of an inverted cross; the alary processes are directed transversely, partially covering the otic area and almost reaching the squamosal; the posterior process of the parasphenoid almost reaches the foramen magnum. In the dorsal view, the long zygomatic ramus of the squamosal presents an elongated and acuminated anterior border that extends towards the level of the maxilla (Fig. 8).

###### Variation

**(Figs 14, 17).** Dorsal surfaces with various shades of brown, with or without darker bands or bars. Darker facial markings such as labial, canthal and interorbital bars are usually present. Dorsal mark is an inverted “V” in the coccygeal region; other patterns include arrangements of thin lines arranged longitudinally. Dorsal surface of hands and feet with irregular brown spots. The fore- and hind limbs are banded with dark brown, separated by light brown interspaces. Flanks with various shades of brown, usually lighter than dorsum. Hind surfaces of thighs with black or dark brown bars and cream interspaces. Groin with orange and sometimes yellowish-white spots on an orange background. Belly brown; sometimes mottled. The belly may have numerous orange to yellowish-white spots. In some specimens, there is a yellowish-white cross on the belly. Iris is golden to bronze with fine black reticulations and a reddish-copper horizontal middle stripe.

###### Call description

**Figure 18.**
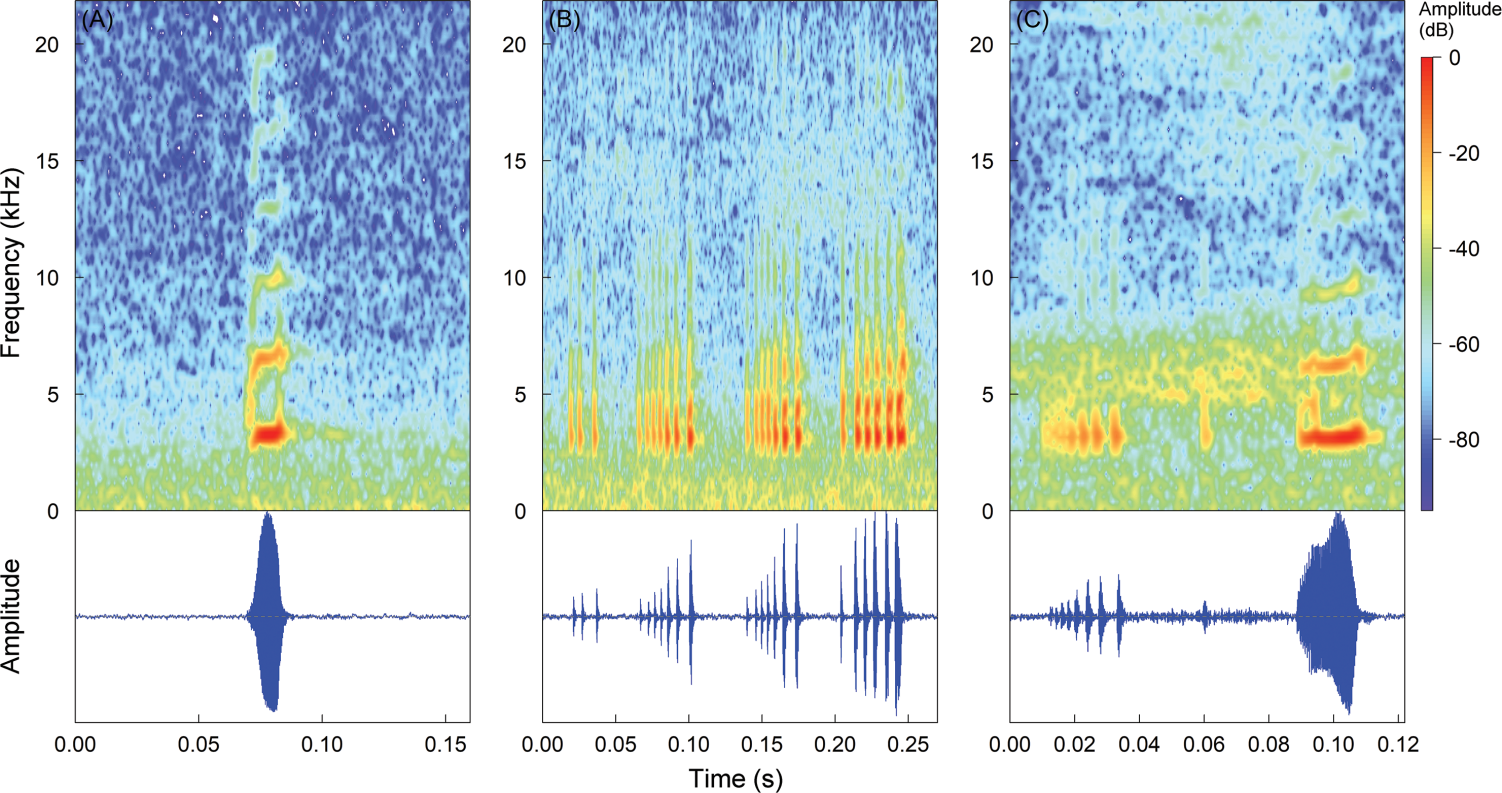
Call of *Pristimantiskayi* sp. nov. (adult male, MZUTI 852). The species has different types of calls, represented as follows: **A** single note call **B** pulsed note calls **C** complex calls (non-pulsed and pulsed notes).

**(Fig. 18).** The description is based on the call of an adult male (MZUTI 852), recorded during the night (21:40 h) by Italo Tapia on 7 June 2012, at the Cordillera de los Guacamayos, Province of Napo, Ecuador. The male was calling from leaf litter at ground level. The air temperature was 12.2 °C. The recording consisted of 21 calls, 37 notes and 856 pulses. The call of *Pristimantiskayi* sp. nov. is composed of the emission of several elements with different characteristics, ranging from single notes to pulsed notes. The call (Fig. 1) has a mean dominant frequency of 3.19 ± 0.05 kHz, with 2–6 partial harmonics in the spectrogram. It has a mean duration of 165.1 ± 130.1 ms. The mean interval between calls is 4565.6 ± 5742.3 ms, emitted at a mean rate of 32.6 ± 23.2 calls/minute. Calls are composed of 1 to 7 notes. Notes have a mean duration of 36.0 ± 18.3 ms. The mean interval between notes is 43.8 ± 36.2 ms, emitted at a mean rate of 15.7 ± 6.1 notes/second. The notes are composed of 2 to 13 pulses. Pulses have a mean duration of 3.9 ± 1.1 ms, with a mean interval between pulses of 4.2 ± 2.8 ms, emitted at a mean rate of 202.2 ± 119.7 pulses/second (Table 4). The call of *Pristimantiskayi* sp. nov. does not present stereotyped notes. Due to their structural elements (i.e. notes–pulses), notes can be classified into three different types: Single note calls (CNS; Fig. 18A), Pulsed note calls (CNP; Fig. 18B) and Complex calls (CNP; Fig. 18C), which contain single (non-pulsed) and pulsed notes in its structure. Temporal and spectral measurements of the different call types are shown in Table 4.

**Table 4.** Spectral and temporal measurements of the call of *Pristimantiskayi* sp. nov. Abbreviations are: DF = Dominant frequency; NH = Number of visible harmonics; 2f0–7f0 = Harmonic frequencies; CD = Call duration; IC Intervals between calls; CR = Call rate; NC = Notes/call; ND = Note duration; IN = Intervals between notes; NR = Note rate; PN = Pulses/Note; PN = Pulse duration; IP = Interval between pulses; PR = Pulse rate. The abbreviations used in the call types correspond to: CNS = Single note calls; CNP = Pulsed note calls; CCO = Complex calls. The abbreviations used in units of measurement correspond to: kHz = kilohertz; ms = milliseconds; s = seconds; /min = per minute; /s = per second.

Parameters	Call	Call Types		
	(general)	Type CNS	Type CNP	Type CCO
CD (ms)	10–463 (165.13 ± 130.06)	10–213 (75.30 ± 83.53)	40–463 (258.29 ± 105.37)	91–189 (114.29 ± 34.30)
IC (ms)	528–32821 (4565.61 ± 5742.25)	–	–	–
CR (/min)	1.82–112.78 (32.61 ± 23.23)	–	–	–
NC	1–7 (2.62 ± 1.68)	1–2 (1.32 ± 0.48)	1–7 (3.76 ± 1.65)	2
ND (ms)	10–111 (36.01 ± 18.32)	10–21 (13.82 ± 2.76)	12–111 (39.85 ± 17.07)	15–35 (21.40 ± 5.28)
IN (ms)	16–184 (43.81 ± 36.18)	134–184 (155.70 ± 17.45)	16–69 (31.94 ± 8.73)	49–150 (70.86 ± 36.13)
NR (/s)	5.13–35.71 (15.68 ± 6.05)	5.13–6.94 (6.14 ± 0.69)	10.42–35.71 (16.95 ± 5.22)	5.75–14.93 (11.55 ± 3.08)
PN	2–13 (5.70 ± 2.19)	–	2–13 (5.55 ± 2.30)	2–9 (6 ± 2.62)
PD (ms)	1–8 (2.95 ± 1.07)	–	1–8 (3 ± 1.07)	1–7 (2.47 ± 1.06)
IP (ms)	0.6–13 (4.19 ± 2.80)	–	0.6–13 (4.32 ± 2.81)	0.6–8 (2.02 ± 1.71)
PR (/s)	66.77–769.23 (202.15 ± 119.72)	–	66.77–769.23 (190.05 ± 108.22)	69.93–769.23 (345.21 ± 157.84)
DF (kHz)	3–3.38 (3.19 ± 0.05)	3–3.38 (3.21 ± 0.07)	3–3.38 (3.19 ± 0.04)	3–3.38 (3.19 ± 0.07)
NH	2–6 (4.09 ± 1.04)	3–7 (5.32 ± 0.91)	3–6 (4.02 ± 1.01)	3–6 (4.07 ± 1.08)
2f0 (kHz)	6–6.75 (6.38 ± 0.09)	6–6.75 (6.46 ± 0.16)	6–6.75 (6.38 ± 0.08)	6–6.75 (6.39 ± 0.11)
3f0 (kHz)	9–10.50 (9.56 ± 0.13)	9–10.13 (9.64 ± 0.22)	9–10.50 (9.55 ± 0.12)	9–10.13 (9.56 ± 0.18)
4f0 (kHz)	12–13.50 (12.75 ± 0.17)	12–13.50 (12.81 ± 0.27)	12–13.50 (12.74 ± 0.15)	12–13.50 (12.80 ± 0.24)
5f0 (kHz)	15–16.88 (15.96 ± 0.24)	15.75–16.88 (16.03 ± 0.27)	15–16.88 (15.94 ± 0.21)	15–16.88 (16.04 ± 0.39)
6f0 (kHz)	18–20.25 (19.16 ± 0.23)	19.13–20.25 (19.25 ± 0.31)	18–19.13 (19.13 ± 0.18)	18.94–20.25 (18.13 ± 0.08)
7f0 (kHz)	22.31	22.31	–	–

###### Distribution and natural history observations.

*Pristimantiskayi* sp. nov. has been recorded from the Cordillera de Guacamayos, Volcán Sumaco (Napo Province) and Cerro Mayordomo, Machay Reserve, Naturetrek Vizcaya Reserve, Leito Reserve (Tungurahua Province) at an elevational range of 2190–3600 m a.s.l. (Fig. 11). Calling males were heard during the day and night. During the night, frogs of *Pristimantiskayi* sp. nov. were found on the forest floor or perched on low vegetation (always below 60 cm from ground level), including shrubs, grasses (*Neurolepis* sp.), ferns and *Selaginella*. The species has been found in primary and disturbed Andean forests, with trees of the genus *Weinmannia*, *Clusia*, *Alnus* and others, of 10–15 metres (Fig. 12).

###### Etymology.

The specific epithet “*kayi*” is a noun in the genitive case and a patronym for Andreas Kay, a German physicist, biologist and friend who spent much of his life documenting and exploring biodiversity, contributing substantially to the conservation of Ecuadorian forests (Fig. 12). Andreas was part of the team that created Fundación EcoMinga’s Dracula Reserva in north-western Ecuador. Additionally, his work shed light on understanding the diversity and endemism patterns of orchids in the Llanganates-Sangay Ecological Corridor, where he passed away in 2019. Some of his amazing photographs can be seen here: https://www.flickr.com/photos/andreaskay/albums/.

## ﻿Discussion

Uncovering the mechanisms behind *Pristimantis* hyperdiversity is challenging. Nevertheless, for any attempt to be successful, we first need a thorough taxon sampling and some insights into the evolutionary relationships of the group. Recent sampling efforts and the mitochondrial phylogeny by Franco-Mena et al. (2023) already provided a first glimpse into the biogeography and speciation patterns of the *Pristimantismyersi* group, now formalized as the subgenus Trachyphrynus. Below, we expand the discussion regarding the newly-described taxa and their most closely-related species.

The two new species described in our study were previously confused either with *Pristimantisfestae* or *P.gladiator*. Lynch and Duellman (1980) reported *P.festae* from the northern versant of Tungurahua Volcano, based on overall similarity. Reyes-Puig et al. (2022) reported these records as an undescribed species (corroborated by the molecular phylogeny produced by Franco-Mena et al. (2023)) and highlighted the importance of microhabitat variation to shape the high diversity and endemism of the area (Jost 2004).

Based on the distribution patterns of the two new species described herein (*P.donnelsoni* sp. nov. and *P.kayi* sp. nov.) and their closest evolutionary relatives (*P.gladiator* and *P.festae*), we can speculate on the relevance of some geographic features of the Andean landscape that may have influenced speciation processes. We note, however, that the relationships amongst these four species are based only on mitochondrial genes and that some of the nodes are short and with bootstrap support that varies from 83% to 100% (Fig. 1). The first interesting pattern to note is that the subgenus Trachyphrynus is restricted to the Andes of Colombia and Ecuador (Franco-Mena et al. 2023), resembling the distribution of other direct-development Andean anurans, such as the genus *Osornophryne* (Páez-Moscoso and Guayasamin 2012). *Pristimantisdonnelsoni* sp. nov. is the southernmost species of *Trachyphrynus* and the only species of the subgenus occurring south of the Pastaza River Valley, an important biogeographic barrier (Lynch and Duellman 1980; Reyes-Puig et al. 2022). The sounds of the calls of *Pristimantisdonnelsoni* sp. nov. are very melodic whistles very similar to the calls of *Pristimantisfestae* (Peracca, 1904) described by Holzheuser and Merino-Viteri (2019). The spectral and temporal values of *P.donnelsoni* sp. nov. relative to *P.festae* vary only slightly, unlike the timbres, which exhibit up to eight series of harmonic frequencies (Holzheuser and Merino-Viteri 2019). It is worth mentioning that the vocalisations of *P.donnelsoni* sp. nov. and *P.festae* are strikingly similar, suggesting a scenario of the retention of ancestral traits.

The distribution of *P.kayi* sp. nov. is limited by the Quijos River to the west; this river seems to be the main barrier separating *P.kayi* sp. nov. and *P.gladiator*. To the south, *P.kayi* sp. nov. is limited by another important barrier, the Pastaza River Valley (Baby et al. 2004). Similar distribution and endemism patterns are observed in other groups of small Andean vertebrates like lizards of the genus *Riama* (Kizirian 1996).

Natural ecosystems inhabited by *P.festae*, *P.gladiator* and the new species are somewhat dissimilar; populations of *P.gladiator* are restricted to montane forests and populations of *P.festae* to higher páramo. On the other hand, *P.kayi* sp. nov. and *P.donnelsoni* sp. nov. are distributed within the montane forest and ecotones with highland paramo ecosystems.

### ﻿Remarks on morphometrics and skull morphology of the *P.gladiator* complex

Skull morphology has proven to be a practical and informative line of evidence for distinguishing cryptic species of amphibians with extremely similar external morphology (Reyes-Puig et al. 2020a, b). Exploring skull osteological characters as a way to resolve the taxonomy of closely-related species is a promising area of research. The main key bones to discriminate species are the sphenethmoid, frontoparietals, squamosal and parasphenoid. A pending issue is having a better assessment of skull variation in relation to ontogeny and sex, a task that is facilitated by CT-scan technology.

## Supplementary Material

XML Treatment for Pristimantis donnelsoni

XML Treatment for Pristimantis kayi

## ﻿Additional information

### Author ORCIDs

Juan Pablo Reyes-Puig https://orcid.org/0000-0002-0730-9356

Miguel Urgilés-Merchán https://orcid.org/0000-0002-4606-2237

Daniela Franco-Mena https://orcid.org/0000-0003-3655-2678

Juan M. Guayasamin https://orcid.org/0000-0003-0098-978X

Diego Batallas https://orcid.org/0000-0002-0068-8146

Carolina Reyes-Puig https://orcid.org/0000-0001-7828-8698

### Data availability

All of the data that support the findings of this study are available in the main text or Supplementary Information.

## ﻿Appendix 1

Examined specimens.

***Pristimantiskayi* sp. nov.**

DHMECN 13835, adult female collected by Luis Recalde on 10 December 2017 in Runacocha Mirador, Río Machay-Río Pastaza, Río Verde, Baños de Agua Santa, Tungurahua, Ecuador (-1.407011, -78.271278; 1600 m alt.). DHMECN 16214 and 16216, adult females collected by Juan Pablo Reyes-Puig in May, 2021 in Vizcaya, Reserva NatureTrek, Ulba, Baños de Agua Santa, Tungurahua, Ecuador (-1.3962203, -78.3941767; 3062 m alt.). DHMECN 14448 and 14484, adult females collected by Mario Humberto Yánez Muñoz, Juan Pablo Reyes-Puig and Daniela Franco-Mena on 2 March 2018 in Cerro Mayordomo, Reserva Ecológica Machay, Río Negro, Baños de Agua Santa, Tungurahua, Ecuador (-1.36866, -78.26928; 2972 m alt.). DHMECN 15242, 15243 and 15244, adult females collected by Kelsey Huisman y Eduardo Peña on 12 December 2019 in Vizcaya, Reserva Nature Trek, Ulba, Baños de Agua Santa, Tungurahua, Ecuador (-1.3962203, -78.3941767; 3150 m alt.). DHMECN 15232, 15234 and 15236, adult females collected by Kelsey Huisman y Eduardo Peña on 24 November 2019 in Cerro Mayordomo, Reserva Ecológica Machay, Río Verde, Baños de Agua Santa, Tungurahua, Ecuador (-1.36866, -78.26928; 2969 m alt.). DHMECN 15249, adult female collected by Juan Pablo Reyes-Puig, Santiago Recalde and Fernanda Flores on 12 December 2019 in Cerro Mayordomo, Reserva Ecológica Machay, Río Verde, Baños de Agua Santa, Tungurahua, Ecuador (-1.36848, -78.26964; 3000 m alt.). DHMECN 18441 and 18442, adult females collected by Juan Pablo Reyes-Puig on 8 July 2022 in Sendero de agua de Güitig, Leyto, Patate, Baños de Agua Santa, Tungurahua, Ecuador (-1.329319, -78.446457; 3300 m alt.). DHMECN 16210, adult male collected with the same data for DHMECN 16212. DHMECN 14449, 14450, 14485 and 14486, adult males collected with the same data for DHMECN 14446. DHMECN 16172, adult male collected by Juan Pablo Reyes-Puig on 10 December 2019 in Cerro Mayordomo, Reserva Ecológica Machay, Río Verde, Baños de Agua Santa, Tungurahua, Ecuador (-1.368666, -78.26923; 2800 m alt.). DHMECN 18440, adult male collected by Juan Pablo Reyes-Puig on 6 July 2022 in Los Mortiños, Leyto, Patate, Baños de Agua Santa, Tungurahua, Ecuador (-1.315235, -78.452793; 3448 m alt.). DHMECN 18443, adult male collected with the same data for DHMECN 18441.

***Pristimantisdonnelsoni* sp. nov.**

DHMECN 4770, adult female collected by Juan Pablo Reyes-Puig and Salomón Ramírez on 31 March 2007 in Pondoa, Baños, Baños de Agua Santa, Tungurahua, Ecuador (-1.437024, -78.442624; 3000 m alt.). DHMECN 4780 and 4784, adult females collected by Juan Pablo Reyes-Puig on 21 March 2007 in Pondoa, Baños, Baños de Agua Santa, Tungurahua, Ecuador (-1.437024, -78.442624; 3240 m alt.). DHMECN 13321, adult female collected by Juan Pablo Reyes-Puig, Esteban Morales and Luis Recalde on 28 December 2016 in Reserva Cerro Candelaria, Río Verde, Baños de Agua Santa, Tungurahua, Ecuador (-1.42952, -78.29962; 3300 m alt.). DHMECN 13831, adult male collected by Mario Humberto Yánez Muñoz, Juan Pablo Reyes-Puig and N. Palacios on 12 April 2017 in Ventanas-Runtún, Volcán Tungurahua, Baños, Baños de Agua Santa, Tungurahua, Ecuador (-1.43799, -78.42506; 3200 m alt.). DHMECN 16175, adult female collected by Juan Pablo Reyes-Puig on 10 November 2019 in Campamento Mandur, Reserva Cerro Candelaria, Río Verde, Baños de Agua Santa, Tungurahua, Ecuador (-1.390224, -78.289318; 3200 m alt.). DHMECN 18159 and 18160, adult females collected by Juan Pablo Reyes-Puig on 28 May 2022 in Bosque Protector Guamag, Ulba, Baños de Agua Santa, Tungurahua, Ecuador (-1.419494, -78.3648; 2800 m alt.). DHMECN 4773, 4775 and 4776, adult males collected with the same data for DHMECN 4780. DHMECN 5086 adult males collected by Juan Pablo Reyes-Puig, Salomón Ramírez and Stalin Cáceres on 11 May 2008, in Bosque Protector Cerro Candelaria, Río Verde, Baños de Agua Santa, Tungurahua, Ecuador (-1.457167, -78.301569; 2850 m alt.). DHMECN 16599, 16602, 16603, 16607, 16609, 16615 and 16617, adult males collected by Juan Pablo Reyes-Puig on 14 August 2018 in Reserva Chamana, Ulba, Baños de Agua Santa, Tungurahua, Ecuador (-1.43432674, -78.39419988; 3125 m alt.). DHMECN 18185, adult male collected by Juan Pablo Reyes-Puig on 14 May 2022 in Finca Palmonte, Río Negro, Baños de Agua Santa, Tungurahua, Ecuador (-1.434871, -78.26291; 2485 m alt.).

***P.festae***

DHMECN 1891 and 1892, adult females collected by Edison Mejía on 06 October 2003 in Embalse Salve Faccha, Oyacachi, El Chaco, Ecuador (-0.231911, -78.153346; 3910 m alt.). DHMECN 9287, 9289, 9291 and 9296, adult females collected by Ligia Cecilia Tobar Suárez in 2009 in Proyecto PRAS, Páramo de Papallacta, Quijos, Napo, Ecuador (-0.352704, -78.175879; 3973 m alt.). DHMECN 11372, 11373, 11374 and 16280, adult females collected by Estella Patricia Bejarano Muñoz on 05 July 2014 in La Virgen, Papallacta, (-0.333398, -78.195149; 4073 m alt.). DHMECN 2459 and 2460, adult males collected with the same data for DHMECN 1891. DHMECN 9286, 9288, 9292, 9293 and 9295, adult males collected with the same data for DHMECN 9287. DHMECN, 16281, 16282 and 16283, adult males collected with the same data for DHMECN 11372. DHMECN 14735 and 14736, adult males collected by Salomón Ramírez on 03 October 2014 in Baeza, Quijos, Napo, Ecuador (-0.47080, -77.88772; 3538 m alt.).

***P.gladiator***

DHMECN 9276, adult female collected by Ligia Cecilia Tobar Suárez in 2009 in Proyecto PRAS, Alrededores de Cuyuja, Quijos, Napo, Ecuador (-0.422013, -78.042368; 2,747 m alt.). DHMECN 12463, 12473, 12478 and 12480 adult female collected by Estella Patricia Bejarano Muñoz, María Pérez Lara, Mario Humberto Yánez Muñoz and Jorge Brito on 09 October 2014 in Proyecto tropiandino OCP, Cuyuja, Quijos, Napo, Ecuador (-0.379253, -78.072986; 2,720 m alt.). DHMECN 9275, 9277 and 9278, adult males collected with the same data for DHMECN 9276. DHMECN 12462, 12469, 12470, 12471, 12474 and 12477, adult males collected with the same data for DHMECN 12463.

## Supplementary materials

Supplementary material 1 Boxplot analysis of morphological data between four related species of *Pristimantis* Data type pdf Explanation note Comparison between four related species of *Pristimantis*, using different morphometrical characters. https://binary.pensoft.net/file/912047This dataset is made available under the Open Database License (http://opendatacommons.org/licenses/odbl/1.0/). The Open Database License (ODbL) is a license agreement intended to allow users to freely share, modify, and use this Dataset while maintaining this same freedom for others, provided that the original source and author(s) are credited.Carolina Reyes-Puig
