# Supplementary figures and images for: ﻿Two new species of terrestrial frogs of the Pristimantisgladiator complex (Anura, Strabomantidae) from the Ecuadorian Andes, with insights on their biogeography and skull morphology
Source: Zookeys. 2023 Sep 26;1180:257–93. doi: 10.3897/zookeys.1180.107333 (PMC10838188; doi:10.3897/zookeys.1180.107333)

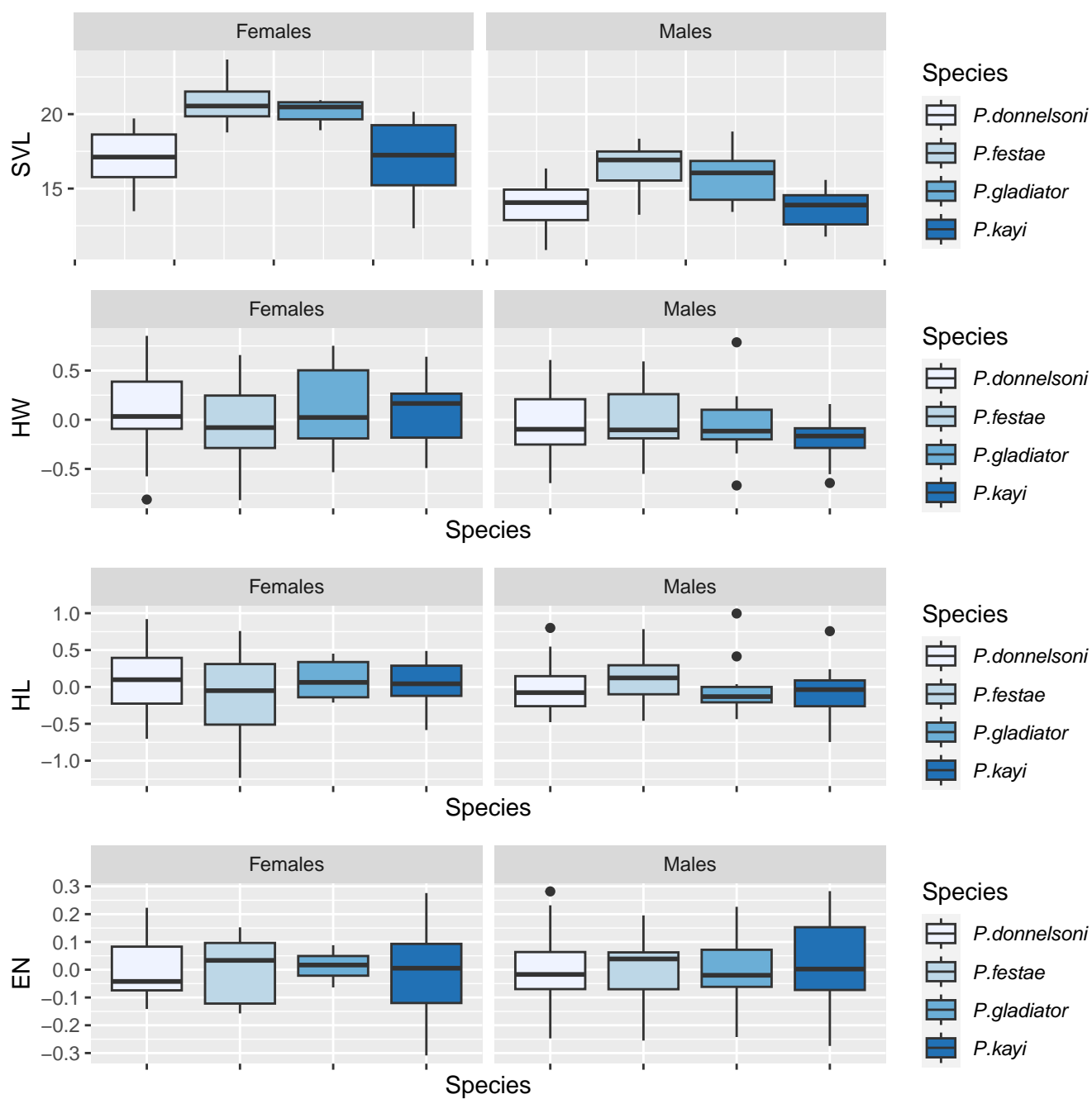

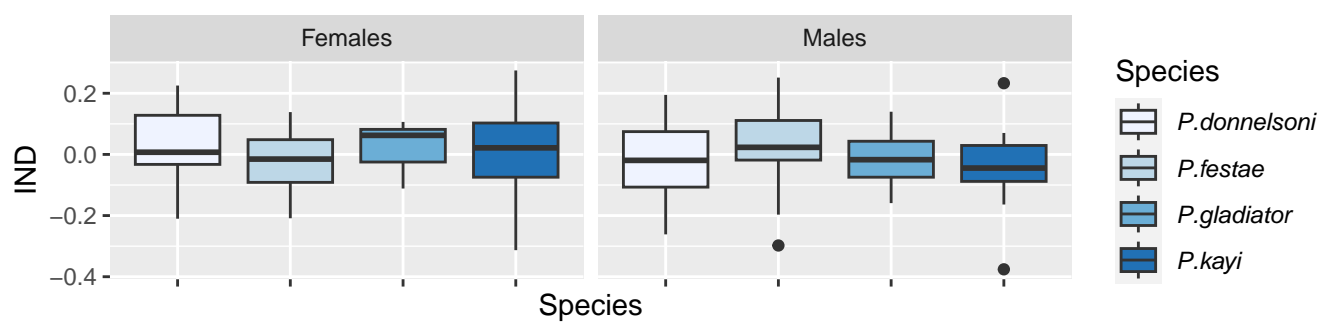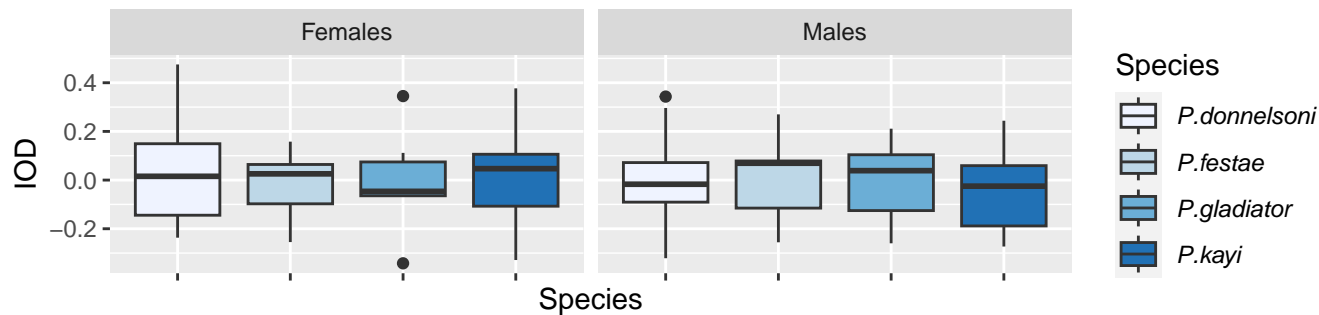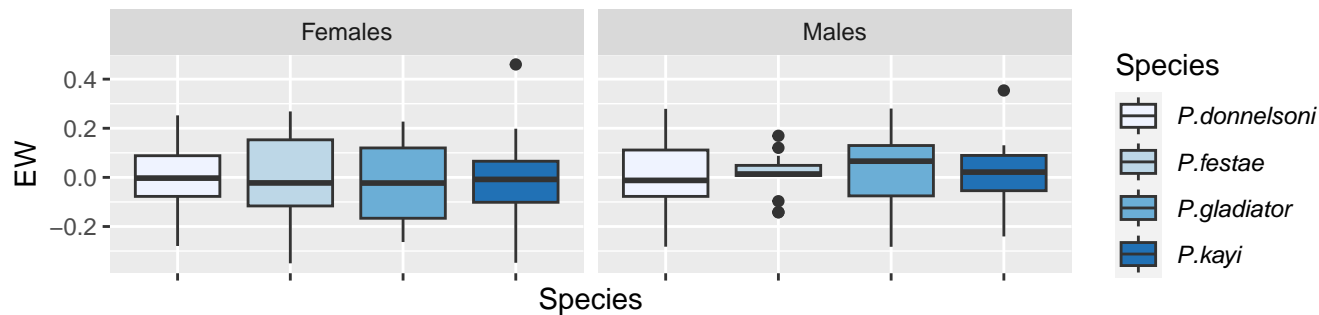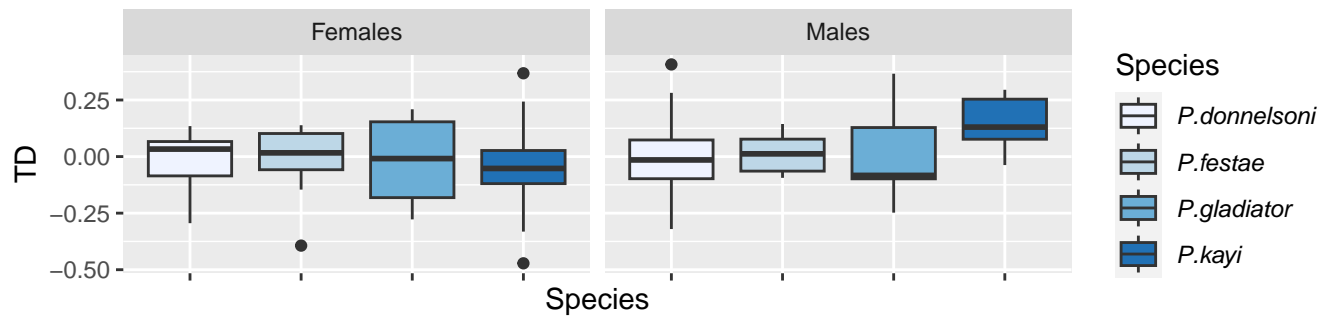

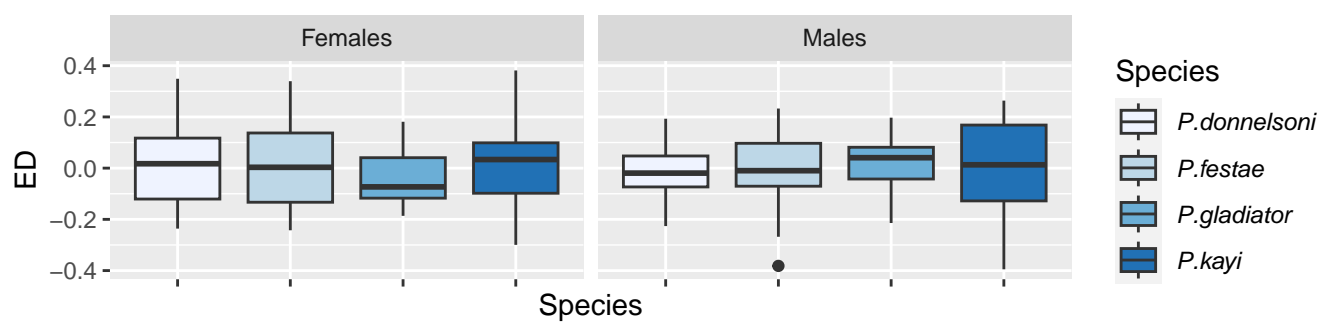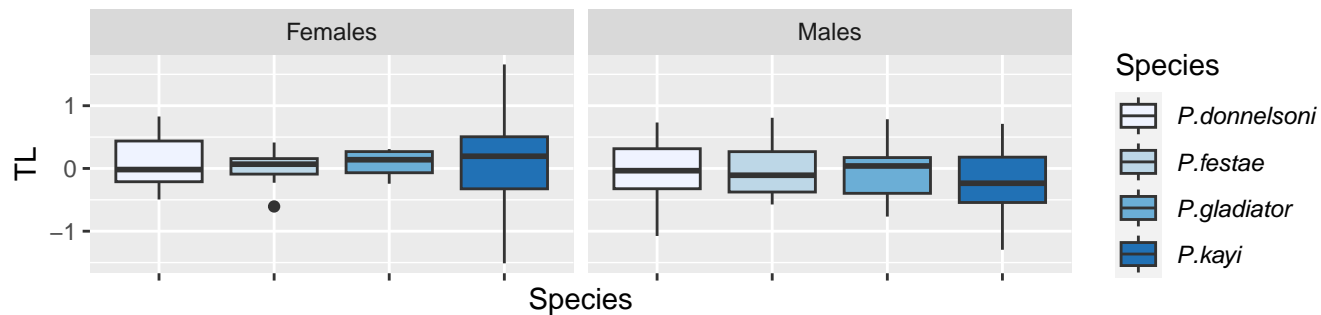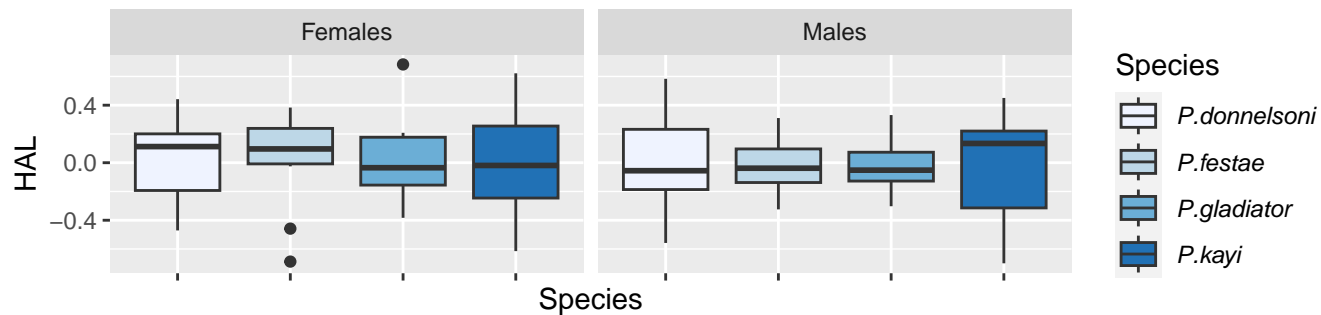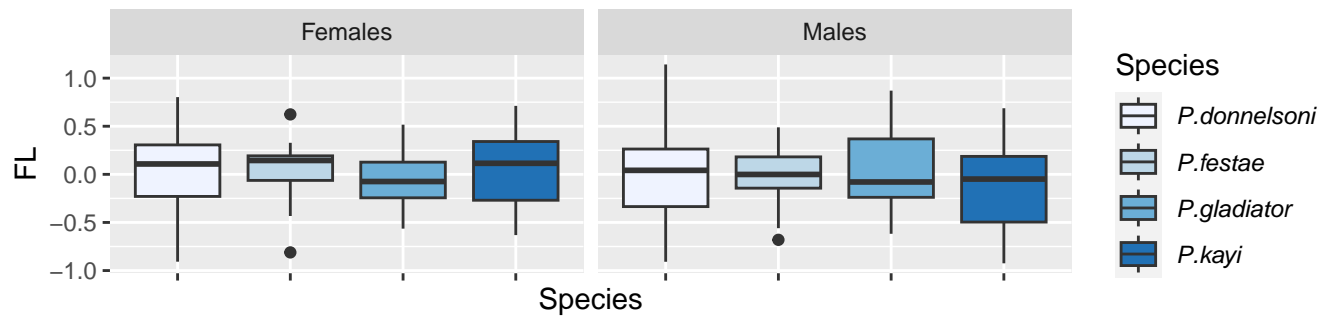

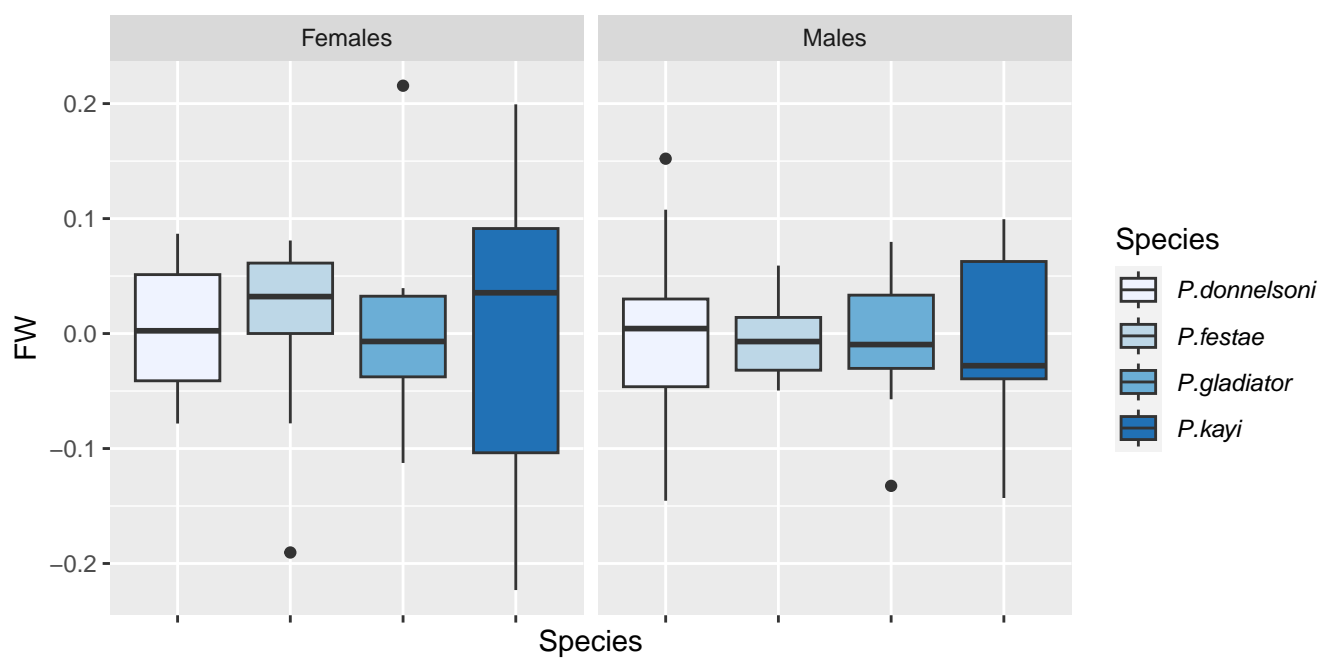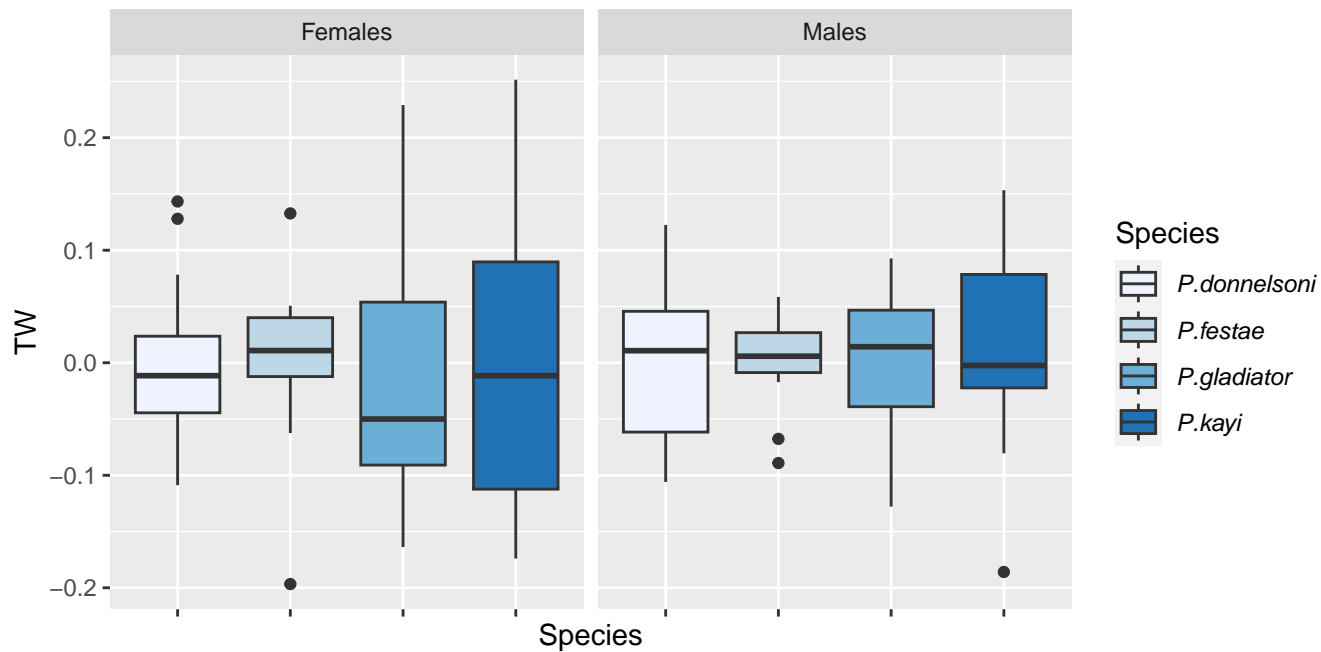

Supplement: Supplementary material 1 — Boxplot analysis of morphological data between four related species of Pristimantis [file zookeys-1180-257_article-107333__-s001.pdf]
